# Novel small molecules targeting ciliary transport of Smoothened and oncogenic Hedgehog pathway activation

**DOI:** 10.1038/srep22540

**Published:** 2016-03-02

**Authors:** Bomi Jung, Ana C. Messias, Kenji Schorpp, Arie Geerlof, Günter Schneider, Dieter Saur, Kamyar Hadian, Michael Sattler, Erich E. Wanker, Stefan Hasenöder, Heiko Lickert

**Affiliations:** 1 Institute of Diabetes and Regeneration Research, Helmholtz Zentrum München, Germany; 2 Institute of Stem Cell Research, Helmholtz Zentrum München, Germany; 3 Institute of Structural Biology, Helmholtz Zentrum München, Germany; 4 Assay Development and Screening Platform, Helmholtz Zentrum München, Germany; 5 Center for Integrated Protein Science Munich at Biomolecular NMR Spectroscopy, Department Chemistry, Technische Universität München, 85747 Garching, Germany; 6 Department of Internal Medicine II, Klinikum rechts der Isar, München, Germany; 7 Technische Universität München, München, Germany; 8 German Center for Diabetes Research (DZD), Germany; 9 German Cancer Consortium (DKTK), Heidelberg, Germany; 10 German Cancer Research Center (DKFZ), Heidelberg, Germany; 11 Neuroproteomics, Max Delbrueck Center for Molecular Medicine, 13125 Berlin, Germany

## Abstract

Trafficking of the G protein-coupled receptor (GPCR) Smoothened (Smo) to the primary cilium (PC) is a potential target to inhibit oncogenic Hh pathway activation in a large number of tumors. One drawback is the appearance of Smo mutations that resist drug treatment, which is a common reason for cancer treatment failure. Here, we undertook a high content screen with compounds in preclinical or clinical development and identified ten small molecules that prevent constitutive active mutant SmoM2 transport into PC for subsequent Hh pathway activation. Eight of the ten small molecules act through direct interference with the G protein-coupled receptor associated sorting protein 2 (Gprasp2)-SmoM2 ciliary targeting complex, whereas one antagonist of ionotropic receptors prevents intracellular trafficking of Smo to the PC. Together, these findings identify several compounds with the potential to treat drug-resistant SmoM2-driven cancer forms, but also reveal off-target effects of established drugs in the clinics.

Hedgehog (Hh) signaling is an evolutionary conserved signaling pathway that plays an essential role in embryonic development^1^,^2^. Postnatal Hh signaling in multiple adult tissues is involved in diverse processes, such as proliferation, differentiation and tissue homeostasis^3^. Dysregulation of the Hh signaling pathway due to mutation in its regulatory components leads to birth defects and various types of cancers^4^. In particular, constitutive activation of the Hh pathway has been identified in basal cell carcinoma (BCC), medulloblastoma (MB) and other sporadic cancer forms^5^,^6^,^7^,^8^,^9^,^10^,^11^.

In vertebrates, Hh signaling is tightly controlled at the primary cilium (PC), a microtubule (MT)-based organelle that emanates from the surface of virtually all mammalian cell types^12^. In the absence of Hh pathway activation, the 12-transmembrane receptor Patched-1 (Ptch1) localizes to the PC and inhibits the activity of the seven transmembrane GPCR Smo by preventing its translocation into the PC^13^. In the presence of ligand, Ptch1 and its ligand move out of the PC, which allows Smo translocation into the PC for Hh pathway activation.

Smo transport into the PC depends on the conserved hydrophobic and basic residue (WR) motif in its cytoplasmic helix VIII^14^. G protein-coupled receptor associated sorting protein (Gprasp) family members are known to directly interact with several GPCRs through the F/WR/K motif in the C-terminal cytoplasmic helix VIII of GPCR for regulation of activity, trafficking and localization^15^,^16^. Our previous study has shown that two novel proteins, Pitchfork (Pifo) and Gprasp2, form a multimeric Smo ciliary targeting complex upon Hh pathway activation (Jung *et al.* submitted). In particular, Gprasp2 binds directly to the WR motif of Smo for regulation of Smo trafficking to the PC, which triggers activation of downstream signaling cascades via regulation of Glioma-associated oncogene (Gli) activity^14^.

Three Gli proteins (Gli1, Gli2, and Gli3) exist in vertebrates. They share a highly conserved zinc finger DNA-binding domain and act as transcriptional regulators^2^,^17^. Gli1 and Gli2 are positive regulators of transcription, whereas Gli3 mainly functions as a repressor of Hh target genes^18^. In response to Hh signaling, Gli proteins get processed and translocate into the nucleus to bind to their consensus Gli-binding site in direct target genes, such as Gli1, Gli2 and Ptch1^19^. This leads to the regulation of a number of target genes involved in multiple cellular processes often associated with malignant transformation^20^,^21^,^22^.

Historically, drug discovery efforts to block constitutive Hh signaling pathway have focused primarily on antagonizing Smo. Several Smo inhibitors are currently in clinical development for a variety of cancer treatments. These include Cyclopamine, a plant-derived steroidal alkaloid, and its derivate Saridegib (IPI-926) and synthetic compounds Vismodegib (GDC-0449), Cur61414 (CAS 334998-36-6), XL-139 (BMS-833923) and Sonidegib (LDE-225)^23^,^24^,^25^,^26^,^27^,^28^,^29^,^30^,^31^,^32^. One of the most advanced drugs in clinical trials, Vismodegib, has been used for the treatment of locally advanced and metastatic BCC and MB. However, there have been disappointing reports for Vismodegib in these tumor entities. The negative outcomes were in part due to *de novo* mutation in Smo (SmoM2 missense mutation that leads to amino acid exchange W535L) or amplification of cell-cycle regulators that leads to tumor resistance^30^,^33^,^34^,^35^. Consequently, alternative approaches to inhibit constitutive Hh pathway activation to overcome drug resistance are urgently needed.

The PC can either suppress or promote tumorigenesis depending on the oncogenic context. BCC and MB are driven by constitutive active Smo, which depends on the PC for pathway activation^36^,^37^. Interestingly, recent studies have shown that small molecule inhibitors of Smo either disrupt slow intracellular trafficking or fast lateral plasma membrane entry of Smo into the PC, thereby blocking Hh pathway activation^38^,^39^. These findings suggest that interfering with molecular machineries that regulate Smo ciliary translocation could be an important alternative approach to block oncogenic Hh pathway activation to overcome tumor resistance.

## Results

### Identification and verification of small molecules to inhibit ligand-independent Hh activation

**Figure 1:**
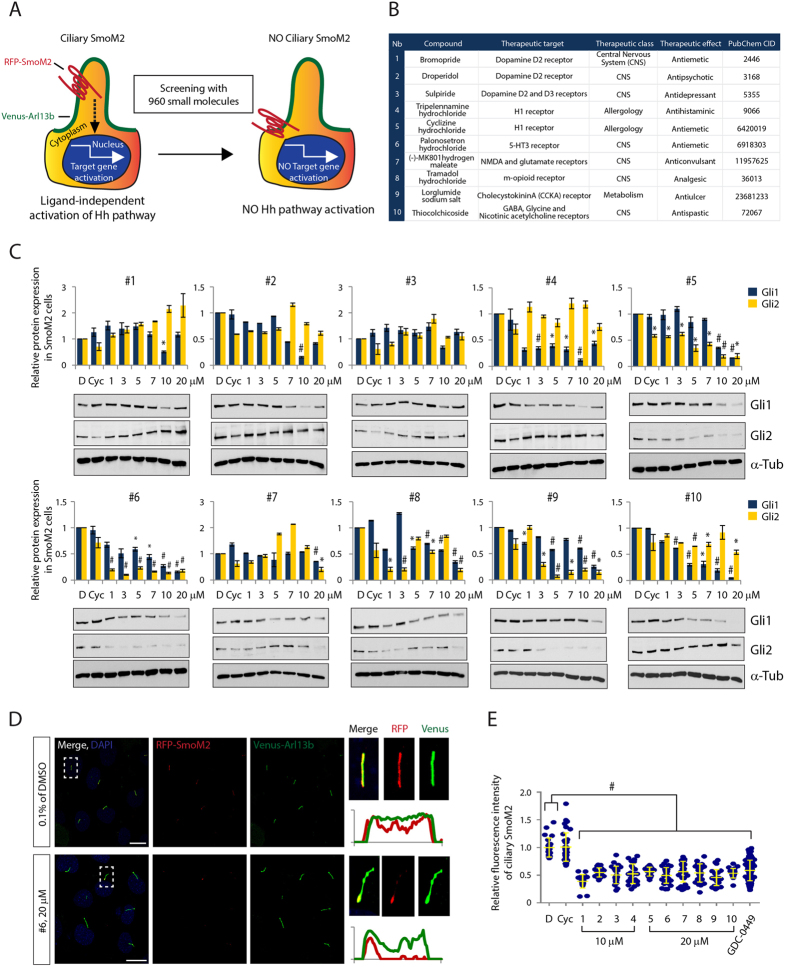
Identification and demonstration of small molecule inhibitors of ciliary accumulation of oncogenic SmoM2 and ligand-independent Hh pathway activation. (**A**) Experimental scheme for high content screening. (**B**) A list of small molecule inhibitors. PubChem CID, http://www.ncbi.nlm.nih.gov/pccompound (**C**) The expression levels of Gli1 and Gli2 protein in LB cells stably expressing RFP-tagged SmoM2 and Venus-tagged Arl13b after treatment with various concentrations of compound 1–10. Graphs indicate quantification of immunoblot data that show the mean fold change of protein normalized to αα-Tubulin (α-Tub) levels. (**D**) Representative images of confocal laser scanning microscopy (CLSM) and (E) quantification of ciliary SmoM2 after compound 1–10 treatment. SmoM2 and Arl13b, PC marker, were stained with antibodies to RFP and Venus respectively. Scale bar = 25 μm. >100 cilia were analyzed per condition. All error bars represent the mean SD of three independent experiments. A one-way ANOVA is used for statistical data analysis. (^#^*p < 0.0001*) D = DMSO, 0.1%; Cyc = Cyclopamine, 10 μM; GDC-0499, 1 μM.

To identify small molecules that inhibit constitutive SmoM2 translocation to the PC resulting in Hh pathway activation, we developed an image-based high content screen (HCS). We used immortalized limb bud (LB) cells stably expressing a Venus-tagged version of Arl13b (Venus-Arl13b), a small GTPase that serves as a marker for the PC membrane, and a red fluorescent protein-tagged SmoM2 (RFP-SmoM2). Without Hh ligand exposure, SmoM2 exhibited constitutive ciliary localization and significantly increased Gli1 and Gli2 target gene expression compared to Smo wild-type (RFP-SmoWT) expressing LB cells (Fig. 1A and Figure S1), validating the assay system.

Next, we established an assay for HCS by optimizing various parameters, such as the seeding density of cells (4 × 10^4^ cells/384well), 18 h induction of ciliogenesis by serum deprivation, 8 μM compound concentration and 24 h incubation for analyzing SmoM2 accumulation at the PC (Figure S2A). Moreover, custom algorithms were developed for automated detection and quantitative multi-parametric image analysis (see Materials and Methods). For the HCS we used a library consisting of 960 small molecule compounds with annotated activities, including Food and Drug Administration (FDA) and European Medicines Agency (EMA)-approved drugs and drug candidates in preclinical or clinical development. After hit validation, we identified 36 small molecules, which abrogated SmoM2 ciliary accumulation. We classified these drugs according to their annotated therapeutic and molecular targets. Strikingly, compounds 1–9 target GPCRs and compound 10 acts as an antagonist of ionotropic receptors, raising the possibility that these compounds might also bind and inhibit the atypical GPCR Smo, thus modulating downstream Hh activation (Fig. 1B and Figure S2B,C). To test this idea, we examined dose-dependent effects of the 10 compounds on SmoM2-dependent signaling in LB cells. All compounds, with the exception of Cyclopamine, prevented Gli1 and Gli2 protein induction at an effective concentration of 10 μM for compounds 1–4 and 20 μM for compounds 5–10 (Fig. 1C). We further confirmed that the compounds inhibited translocation of SmoM2 to the PC at their effective concentration and used the known Smo antagonists, Vismodegib and Cyclopamine as controls (Fig. 1D,E and Figure S3). These results indicate that several FDA-approved compounds can block constitutive SmoM2-mediated PC translocation and Hh signaling activation.

### All identified small molecules inhibit ciliary localization of endogenous Smo and Hh pathway activation

**Figure 2:**
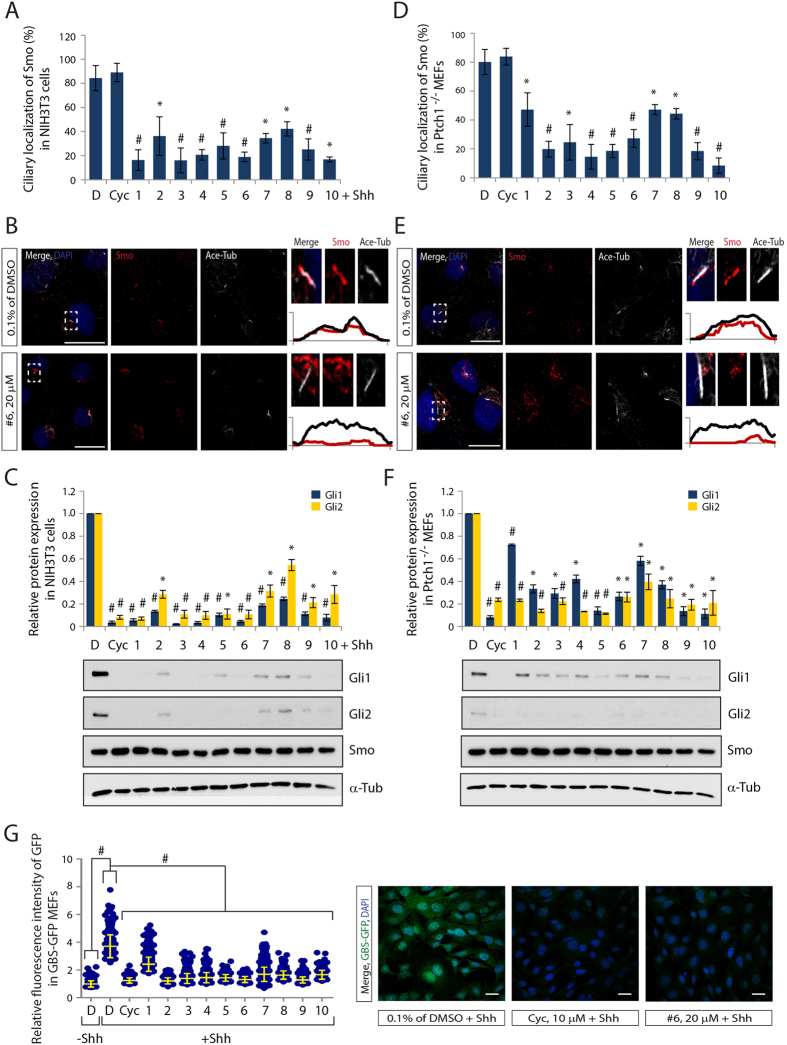
All small molecules effectively inhibit endogenous Smo localization to PC and Hh target gene activation. (**A,D**) Quantification of ciliary Smo, (**B,E**) representative images of CLSM and (**C,F**) the expression levels of Gli1, Gli2 and Smo protein after treatment with compound 1–10 in NIH3T3 cells (**A–C**) or Ptch1^−/−^ MEFs (**D–F**). Smo and PC were stained with antibodies to Smo and Acetylated-Tubulin (Ace-Tub) respectively. Graphs indicate quantification of immunoblot data that show the mean fold change of protein normalized to α-Tub levels. (**G**) Quantification of fluorescence intensity and representative CLSM images of GBS-GFP MEFs after treatment with compound 1–10. The Gli activities were visualized by GFP staining. Scale bar = 25 μm. > 100 cilia were analyzed per condition. All error bars represent the mean SD of three independent experiments. A two tailed unpaired *t-test* (**A,C,D,F**) and a one-way ANOVA (**G**) are used for statistical data analysis. (*p < 0.01, ^#^p < 0.001) D = DMSO, 0.1%; Cyc, 10 μM; compound 1–4, 10 μM; compound 5–10, 20 μM.

The ability of several compounds to inhibit ciliary SmoM2 localization and Hh pathway activation in an overexpression system raised the question of whether these drugs also target wild-type Smo. To test this idea we used several established model systems: First, NIH3T3 cells containing all the necessary components to respond to Hh ligands induction, and second, Ptch1^−/−^ mouse embryonic fibroblasts (MEFs), which show ligand-independent Smo ciliary localization and constitutive pathway activation. Treatment of compounds with their effective concentration significantly inhibited ciliary localization of endogenous wild-type Smo in both cell lines (Fig. 2A,B,D,E and Figure S4). Moreover, Gli1 and 2, but not Smo protein levels, were blocked by compound treatment, comparable to Cyclopamine treatment in ligand exposed NIH3T3 cells and in non-ligand exposed Ptch1^−/−^ MEFs (Fig. 2C,F). The cell viability assay in NIH3T3 cells additionally revealed that all tested compounds did not show cytotoxicity (Figure S5). This demonstrates that the inhibitory effect of the compounds on Hh pathway is not a consequence of cytotoxic effect.

Furthermore, we tested whether compounds could inhibit Gli activity using MEFs generated from a transgenic Hh reporter mouse, in which eight concatemerized Gli transcription factor binding sites upstream of a minimal promoter drive green fluorescent protein (GFP) expression (GBS-GFP)^40^. As expected, Hh treatment enhanced the GBS-GFP bulk fluorescent intensity. In contrast, treatment with all tested compounds significantly reduced the relative GBS-GFP bulk fluorescence intensity, suggesting efficient inhibition of Gli-mediated reporter gene activation (Fig. 2G). We further determined the inhibitory concentration (IC50) of the compounds for transcription of GBS-GFP reporter activity and Ptch1 activation, demonstrating the inhibitory effect of the compounds on the transcriptional activation of Hh target genes (Figure S6).

Suppressor of fused (Sufu) is a negative regulator of Hh pathway that binds directly to Gli proteins, repressing Gli transcriptional activation^41^. Sufu acts downstream of Smo, and Sufu^−/−^ MEFs show constitutive Gli activity in a Smo independent manner^42^. To test whether the compounds act at the level of or downstream of Smo, we examined Gli transcriptional activity in compound-treated Sufu^−/−^ MEFs. None of the compounds were able to block Gli1 expression in these MEFs suggesting that the compounds act at the level of Smo to prevent target gene activation (Figure S7).

### Small molecules inhibit Hh pathway activation by distinct mechanisms

**Figure 3:**
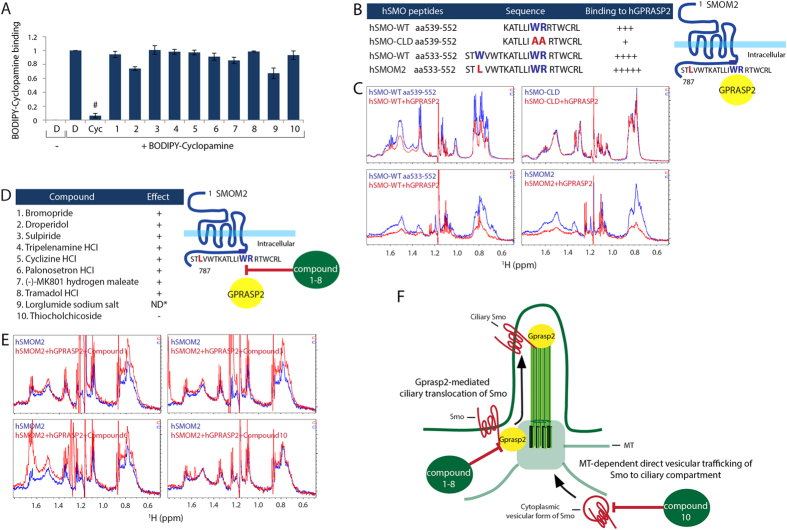
Identification of novel mechanisms of known small molecules in Hh signaling pathway. (**A**) Compound competition assay with fluorescence BODIPY-Cyc for Smo binding in HEK293T cells as monitored by FACS analysis. All error bars represent the mean SD of three independent experiments. A two tailed unpaired *t-test* is used for statistical data analysis. (^#^*p < 0.001*) D = DMSO, 0.1%; BODIPY-Cyc, 20nM; Cyc, 10 μM; compound 1–10, 10 μM (**B**) The left panel of the table presents NMR data summaries of binding specificity of hGPRASP2 to different hSMO peptides harboring the helix VIII. The right panel shows a schematic illustration of SmoM2 and Gprasp2 interaction. (**C**) Selected aliphatic region on ^1^H NMR spectra of the different hSMO peptides were acquired before (blue line) and after addition of full-length hGPRASP2 (red line). (**D**) The left panel of the table shows NMR data summaries of compound interference of hGPRASP2 binding to hSMOM2. The right panel presents a schematic illustration of compound 1–8 effects on hSMOM2 and hGPRASP2 interaction. (**E**) Selected aliphatic region on ^1^H NMR spectra of the hSMOM2 peptide were acquired before (blue line) and after addition of full-length hGPRASP2 with compounds (red line). Note that compound 9 caused hSMOM2 peptide and hGPRASP2 protein precipitation at the experimental concentration. Therefore, it was not able to determine the compound action on the SmoM2 binding to Gprasp2. (ND*, not determined) (**F**) Hypothetical model of compounds’ action on Smo trafficking to the PC. Compounds 1–8 directly inhibit Smo and Gprasp2 interaction that is important for ciliary Smo localization. Compound 10 inhibit direct vesicular trafficking of Smo to ciliary compartment by disrupting MT network (Supplemental Fig. 5E).

To understand how the compounds interfere with the Smo trafficking machinery, we first tested whether the compounds directly bind to Smo and thereby antagonize Smo activity. Cyclopamine is known to directly bind to the heptahelical bundle of Smo^43^. Therefore, a competition assay was performed in Smo-expressing HEK293T cells using BODIPY-Cyclopamine, a fluorescent Cyclopamine derivate^23^. Despite a high concentration (10 μM) of all compounds being applied, none of the compounds except Cyclopamine itself influenced the BODIPY-Cyclopamine binding to Smo. This argues that the compounds do not bind to the heptahelical bundle of Smo (Fig. 3A).

Next, we tested if the compounds interfere with the Smo transport machinery to the PC. We have recently identified that Gprasp2 as an essential component of the Hh pathway, which is required for Smo ciliary trafficking and Hh pathway activation (Jung *et al.* submitted). Particularly, Gprasp2 binds directly to the conserved hydrophobic and basic residue ciliary targeting motif (CTM) of Smo required for ciliary transport (Jung *et al.* submitted)^14^. Interfering with this protein-protein interaction might offer a potential molecular target for drug action. To test this hypothesis, we first performed NMR studies to verify the direct interaction between human GPRASP2 (hGPRASP2) and human SMO (hSMO) peptides. hSMO-CLD (aa539–552) with a mutation in the CTM, which has an impact on GPRASP2 binding affinity, served as a negative control (Jung *et al.* submitted). The longer hSMO-WT (aa533–552) peptide has a higher affinity to hGPRASP2 than the short hSMO-WT (aa539–552) peptide, whereas hSMO-CLD (aa539–552) showed no obvious affinity for hGPRASP2 binding (Fig. 3B,C). Moreover, the hSMOM2 peptide showed the strongest binding affinity to hGPRASP2 in the assay (Fig. 3B,C), suggesting that the W535L mutation of SMO does not interfere with SMO-GPRASP2 protein interaction, but rather increases affinity to GPRASP2. We further tested direct influence of the 10 compounds on the interaction between hSMOM2 and hGPRASP2. Compound 9 could not be tested due to peptide and protein precipitation at the tested concentrations. Interestingly, all compounds interfered with the hSMOM2-hGPRASP2 interaction, except compound 10 (Fig. 3D,E). Co-immunoprecipitation (co-IP) studies in HEK293T cells confirmed the NMR observations that hGprasp2 interacts with mouse Smo (mSmo-WT) and mSmoM2 in a CTM-dependent manner, and all compounds, with the exception of compound 10, interfere with the binding of hGprasp2 to mSmo-WT and mSmoM2 (Figure S8A,B). Together, these results suggest that compound 1–8 directly affect the binding of SMOM2 to GPRASP2, thereby preventing ciliary target complex formation and accumulation at the PC (Fig. 3F).

The PC is a MT-based cellular organelle that originates from the basal body, which is the mother centriole of the centrosome. Hh signaling and components of the Hh signal transduction pathway required PC, and mutations that affect ciliogenesis impair signal transduction^44^. To investigate the specificity of drug action on Smo ciliary localization, but not on ciliogenesis *per se*, we examined PC in NIH3T3 cells after compound treatment. Immunofluorescent staining to visualize the MT-based axoneme of the PC (anti-Acetylated Tubulin) and the centrioles (anti-Pericentrin) showed no significant changes on centriole number, PC number or PC length after drug treatment (Figure S9A,B,C).

Finally, we investigated the drug effects on the MT cytoskeleton. Ciliary targeting proteins move along MTs from the cytoplasm to the PC compartment through interaction with Tubulin and Tubulin- associated motor proteins^45^. Several MT inhibitors that interfere with this process act as Hh pathway antagonists^26^,^46^. We observed severe disruptions of the MT network with centriole disengagement and cytoplasmic mislocalization of Smo only after treatment with compound 10 (Thiocolchicoside), a semi-synthetic derivate of colchicine, which is a known inhibitor of MT polymerization (Figure S9A,D). In the Hh pathway, Smo is stored in cytoplasmic vesicles near the base of the PC^47^. After disruption of the MT network by compound 10, subcellular localization of Smo in cytoplasmic vesicles was dispersed all over the cytoplasm into the cell periphery, which very likely affected Smo ciliary trafficking (Figure S9E).

### Effect of small molecules on oncogenic ciliary SmoM2-mediated Hh signaling in pancreatic cancer

**Figure 4:**
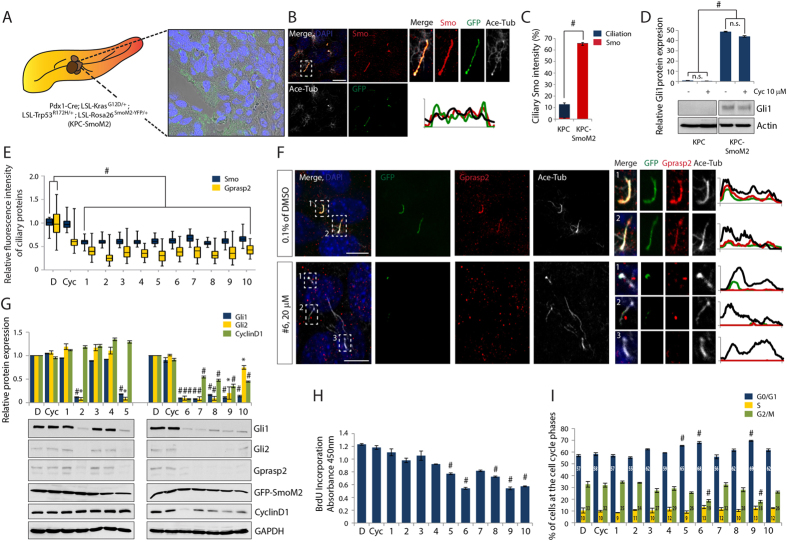
Small molecules’ effect on ciliary SmoM2-mediated constitutively active Hh signaling and PDAC tumor growth. Representative CLSM images of (**A**, Movie S1) tissue specimen and (**B**) primary PDAC cells from a KPC-SmoM2 (*Pdx1-Cre; LSL-Kras*^*G12D/*+^*; LSL-Trp53*^*R172H/*+^*; LSL-Rosa26*^*SmoM2−YFP/*+^) mouse strain. (**A**) YFP-tagged SmoM2 is visualized with GFP antibody and the GFP signals display the localization of SmoM2 on invasive PDAC region. (**B**) Immunostaining with antibodies against Smo, GFP, and Ace-Tub show that the accumulation of SmoM2 along the PC of primary PDAC cells. (**C**) Graphs indicate quantification of ciliation and ciliary Smo in primary KPC-SmoM2 cells compared to primary KPC cells. (**D**) Gli1 protein expression levels after treatment with or without Cyc in primary KPC or KPC-SmoM2 PDAC cells. Graphs indicate quantification of immunoblot data that show the mean fold change of Gli1 protein normalized to Actin levels. Note that isolated primary KPC cells were derived from a *Pdx1-Cre; LSL-Kras*^*G12D/*+^*; LSL-Trp53*^*R172H/*+^ mouse strain. (**E**) Quantification of fluorescence intensities of ciliary Smo and Gprasp2 and (**F**) representative CLSM images of primary KPC-SmoM2 cells after treatment of compound 1–10. PC are visualized with an antibody against Ace-Tub, and SmoM2 and Gprasp2 are visualized with antibodies to GFP and Gprasp2 respectively. (**G**) The expression levels of Gli1, Gli2, Gprasp2, SmoM2 and Cyclin D1 protein after treatment with compound 1–10 on Primary KPC-SmoM2 cells. Graphs indicate quantification of immunoblot data that show the mean fold change of protein normalized to GAPDH levels. (**H**) Statistical analysis of BrdU incorporation and (**I**) Statistical evaluation of cell cycle phases determined by flow cytometry using propidium iodide (PI) DNA staining after compound 1–10 treatment on primary KPC-SmoM2 cells. Scale bar = 10 μm. >100 cilia were analyzed per condition. All error bars represent the mean SD of three independent experiments. A two tailed unpaired *t-test* (**C,D,G,H**,**I**) and a one-way ANOVA (**E**) are used for statistical data analysis. (^*^p < 0.01, ^#^p < 0.001) D = DMSO, 0.1%; Cyc, 10 μM; compound 1–4, 10 μM; compound 5–10, 20 μM.

Due to the recent implication of Hh signaling in pancreatic ductal adenocarcinoma (PDAC) initiation and progression^48^, we generated a new PDAC model. We crossed the well-characterized KPC mice harboring both conditional *Kras*^*G12D*^ and *p53*^*R172H*^ alleles^49^,^50^ to SmoM2 mice carrying a conditional, constitutive active SmoM2 allele fused to YFP^51^. The additional SmoM2 oncogenic activation in KPC mice (hereafter KPC-SmoM2; *Pdx1-Cre; LSL-Kras*^*G12D/*+^*; LSL-Trp53*^*R172H/*+^*; LSL-Rosa26*^*SmoM2−YFP/*+^) leads to a significantly shorter life expectancy (median 75.5 d, n = 7) than that of KPC mice (median 160 d, n = 61). All tumors were analyzed for YFP expression by immunohistochemistry using an antibody to GFP that also detects the YFP derivate. We observed strong GFP signals in PC structures in the invasive PDAC lesions (Fig. 4A) and confirmed SmoM2 localization in the PC of pancreatic tumor cells by co-localization studies (Fig. 4B,C). Furthermore, significantly higher Gli1 protein levels were observed in the KPC-SmoM2 model compared to the KPC model, indicating oncogenic activation of the Hh signaling pathway (Fig. 4D). Interestingly, the Gli1 protein expression in these PDAC cells was not affected by Cyclopamine treatment, which is in line with the results observed in SmoM2 expressing LB cells (Fig. 1). In contrast, all tested compounds act on SmoM2 to prevent Hh pathway activity. We therefore investigated the compounds’ inhibitory effects on oncogenic Hh pathway in the KPC-SmoM2 model.

First we examined whether the compounds prevent SmoM2-mediated Hh pathway activation by disrupting SmoM2 trafficking to the PC of primary PDAC cells. Treatment of compounds 1–10 in primary PDAC cells derived from the KPC-SmoM2 strain significantly inhibited ciliary SmoM2 and Gprasp2 translocation, suggesting that compound treatment interferes with Smo ciliary targeting complex formation (Fig. 4E,F and Figure S10). Furthermore, treatment with compounds 2 or 5–10 blocked Gli1 and Gli2 target gene activation, in contrast to compounds 1, 3, 4, and Cyclopamine treatment (Fig. 4G). Notably, we also observed that compound treatment led to obvious changes in total protein levels of Gprasp2, whereas Smo levels remained unchanged (Fig. 4G), confirming that Gprasp2 is a downstream target of Hh signaling (Jung *et al.* submitted).

In addition to Hh activation, mutation of Kras and p53 promotes cell-cycle progression through the induction of the G1 to S phase regulator CyclinD1^52^. To determine whether the Gprasp2-SmoM2 antagonists inhibit cell cycle progression, we analyzed the expression of CyclinD1 after compound treatment. The CyclinD1 protein level was significantly reduced by 5 of the tested compounds, and especially by compound 6 (Palonosetron hydrochloride) and compound 9 (Lorglumide sodium salt) (Fig. 4G), which was confirmed by BrdU incorporation assays (Fig. 4H).

We further determined the DNA content of compound-treated primary tumor cells using flow cytometry to test whether tumor cell proliferation is arrested at a particular cell cycle stage. After treatment with compounds 6 and 9 we observed a progressive accumulation of cells in G0 to G1 phase and significantly less cells in G2 to M phase, suggesting a block or delay in G1 to S phase transition in primary PDAC cells (Fig. 4I).

To confirm the specificity of compounds on PDAC tumor cell growth with ciliary Smo-dependent Hh pathway activation, we tested the effects of compounds on primary PDAC cells isolated from the KPC mouse strain. The primary PDAC cells did show rare ciliated cancer cells without Smo accumulation at the PC and Hh pathway activation, but with high expressions of CyclinD1 resulting from different mutations of cell cycle regulators (Figure S11B,C). None of the compounds altered the expression levels of CyclinD1 (Figure S11C) or the cell cycle progression determined by the BrdU incorporation assay (Figure S11D), demonstrating their specific interference with the Hh pathway.

**Figure 5:**
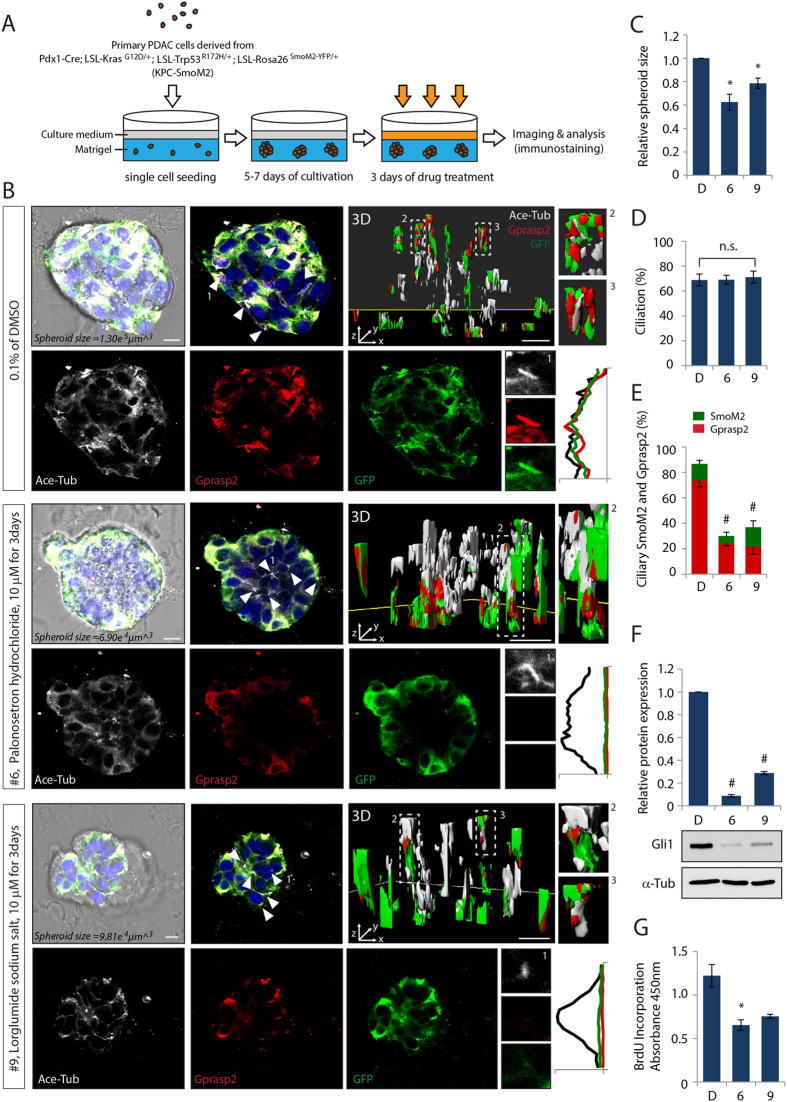
Compound 6 and 9 effect on PDAC tumor spheroids growth by antagonizing ciliary SmoM2-mediated Hh pathway activation. (**A**) Illustration of experimental scheme to generate 3D tumor spheroids and for compounds administration. (**B**) Representative CLSM images and (3D) 3D reconstruction of whole-mount staining of spheroids after treatment with compound 6 or 9. PC are visualized with an antibody against Ace-Tub, and SmoM2 and Gprasp2 are visualized with antibodies to GFP and Gprasp2 respectively. Quantification of (**C**) size, (**D**) ciliation, and (**E**) ciliary SmoM2 and Gprasp2 of spheroids treated with compound 6 or 9. (**F**) Gli1 protein expression levels in spheroids treated with compound 6 or 9. Graphs indicate quantification of immunoblot data that show the mean fold change of protein normalized to α-Tub levels. (**G**) Statistical analysis of cell cycle proliferation of spheroids determined by BrdU incorporation assay after compound 6 or 9 treatment. Scale bar = 10 μm. >100 cilia and tumor spheroids were analyzed per condition. All error bars represent the mean SD of three independent experiments. A two tailed unpaired *t-test* is used for statistical data analysis. (*p < 0.01, ^#^p < 0.001).

We further evaluated the ability of these two compounds to inhibit ciliary localization of the endogenous SmoM2 complex and oncogenic Hh target gene activation using an *in vitro* tumor spheroid culture system (Fig. 5). First, we determined the effective concentration and inhibitory concentration (IC50) of compounds 6 and 9 for Hh pathway and cell proliferation, respectively (Figure S12). Although the maximal inhibitory effects were achieved at high concentrations of compounds, we decided to use the compounds at low doses of 10 μM for long-term drug treatment in order to avoid any possible unspecific cytotoxicity. The growth inhibitory effects of the spheroids derived from primary PDAC cells of KPC-SmoM2 mice were then analyzed after 5–7 days of spheroid cultivation with administration of the compounds for 3 days given with a dose interval of 24 hours (Fig. 5A). Bright field imaging analysis was used to measure the major and minor axial length of the imaged 3D tumor spheroids and to calculate the volume of each individual tumor spheroid. The spheroids treated with compounds showed 20–40% reduction in the tumor spheroid mass (Fig. 5C).

In order to investigate whether the growth of tumor spheroids is dependent on ciliary SmoM2-induced Hh pathway activation, we analyzed Smo and Gprasp2 translocation to the PC and Gli1 protein expression as an indicator for Hh pathway activation. *Whole mount* immunostaining combined with 3D visualization and reconstruction of Z-stack images of the spheroids revealed that the compounds inhibited Gprasp2 and Smo protein accumulation at the cilia *in situ*, but caused no changes in ciliogenesis (Fig. 5B,D,E). Furthermore, activation of Gli1 is almost abolished in compound-treated spheroids, indicating effective inhibition of Hh signaling (Fig. 5F). Finally, we determined the ability of the compounds to inhibit cell cycle progression. The incorporation of BrdU was decreased in cells treated with compounds 6 or 9, indicating reduced cell proliferation rates that correlate with the size of the tumor spheroid mass (Fig. 5G).

Hh signaling promotes cell proliferation through the regulation of its direct target genes, i.e. CyclinD, CyclinE, and Myc, which modulate the G1 to S phase cell cycle transition^21^. Our findings suggest that the inhibitory effect of compounds on Smo activity blocks the transcriptional regulation of CyclinD1 involved in cell cycle progression, which then results in the growth arrest of tumor cells.

## Discussion

Inappropriate activation of Hh signaling is involved in different stages of carcinogenesis in various types of cancer, including BCC, MB and PDAC^53^. Vertebrate Hh signaling depends on the PC and ciliary enrichment of Smo in response to Hh ligand binding to Ptch1. Therefore, pharmacological blockade of ciliary Smo has emerged as a potential anti-cancer therapeutic strategy. However, therapeutic challenges still remain in cases where tumors acquire resistance to Smo antagonists by gaining oncogenic mutations that mediate ligand-independent Hh activation.

To identify novel drugs that block oncogenic SmoM2 we have developed a high-throughput image-based screen. Strikingly, compounds targeting GPCRs emerged as a new class of drug candidates that specifically target the atypical GPCR Smo (Fig. 1). All identified compounds suppress accumulation of wild-type Smo and oncogenic SmoM2 at the PC as well as aberrant Hh activation (Figs 1 and 2).

Previously, we have identified a novel heterotrimeric Smo-Gprasp2-Pifo complex that is regulated by Hh signaling and required for ciliary trafficking of Smo and Hh pathway activation (Jung *et al.* submitted). In particular, Gprasp2 itself is a downstream Hh target and enhances Smo transport and Hh activation, and thus acts to sustain high Hh activation levels (Jung *et al.* submitted). Strikingly, both the mRNA and protein expression levels of GPASP2 are significantly elevated in most Hh-associated cancers (http://explore.pediatriccancergenomeproject.org; http://www.proteinatlas.org). Therefore, it seems very attractive to target the Smo-Gprasp2 ciliary targeting complex to suppress ligand-independent Hh activation and disrupt a feed-forward loop of constitutive Hh activation.

In this study, we demonstrated that a peptide from human SMOM2 directly interacts with human GPRASP2 protein for trafficking to the PC (Fig. 3). Interaction between SMO and GPRASP2 depends on a hydrophobic and basic residue ciliary targeting motif (WR) in the cytoplasmic helix VIII, which is conserved in GPCRs (Jung *et al.* submitted)^14^,^16^. Interestingly, compounds 1–8, which effectively interfere with SMO-GPRASP2 interaction, exert their biological effects by targeting GPCR receptors, such as Dopamine D2 and D3, Histamine H1, Serotonin 5-HT3, and μ-opioid receptors, or by targeting ionotropic N-Methyl-D-aspartate (NMDA) receptors, which contain the conserved hydrophobic and basic residue motif (F/WR/K) in the cytoplasmic helix VIII (Table S1)^14^ suggesting that this helix may be relevant for their mechanism of action. Although there is no crystal structure information of these compounds bound to their respective GPCR targets, it is plausible that compounds 1–8 are interfering with GPRASP2 binding to SMO helix VIII via the WR motif. In fact, amino acid sequence alignment and analysis (Table S1) showed that targets of compounds 9 and 10 do not contain the F/WR/K motif in their cytoplasmic helix region, indicating that this region is relevant to the drug action, and explaining why compound 10 did not show an effect in the NMR experiments shown in Fig. 3. Therefore, compounds 9 and 10 may have their inhibitory actions on Smo through other indirect mechanisms. Thus, compounds 1–8 presumably play a role in the regulation of the WR motif mediated SMO and GPRASP2 interaction, which results in the disruption of the ciliary targeting complex to antagonize Hh signaling.

In our study we were not able to test direct interference of compound 9 (lorglumide sodium salt), an inhibitor of cholecystokinin A (CCKA) receptor, on SMO-GPRASP2 interaction. However, it is interesting to note that CCKA receptor activation leads to stimulation of the cytosolic phospholipase pathway resulting in activation of PKCs^54^,^55^,^56^. Recent studies have demonstrated a cross-talk between the Hh and PKC signaling pathway. Hh signaling induces the expression of atypical PKC (aPKC), which in turn promotes phosphorylation and activation of Smo in *Drosophila*^57^. Moreover, this study revealed that aPKC regulates the activity of Cubitus interruptus (Ci) through phosphorylation of the Zn finger DNA-binding domain, demonstrating the positive role of aPKC in Hh signaling. In vertebrate, aPKC is accumulated along the cilia in Hh-dependent BCC^34^. aPKC also acts downstream of Smo to phosphorylate Gli to regulate its transcriptional activation^34^. Given the obvious cross-talk between these signaling pathways, it is tempting to speculate that activation of aPKC by the CCKA receptor antagonist has an effect on Smo accumulation in the PC and activation of downstream signaling.

There are several antagonists for Hh signal transduction such as JK184, Nocodazole and Colchicine that regulate the Gli transcription by acting on MT depolymerization^26^,^58^,^59^. We identified Thiocolchicoside, a semi-synthetic derivative of the naturally occurring compound Colchicoside, as a potent inhibitor of Hh signaling. Thiocolchicoside possesses a molecular structure similar to Colchicine, which binds to Tubulin and induces the depolymerization of MT, which further supports the notion that Hh signal transduction depends on the MT cytoskeleton.

One of the major roles of MT is regulation of intracellular transport, including trafficking of ciliary targeting proteins from the cytosol and Golgi apparatus to the ciliary compartment and *vice versa*. Surprisingly, none of the drugs affecting MT depolymerization disrupt the stability of ciliary axonemal MTs, but do have an effect downstream of ciliary events such as Gli transport from the cytoplasm into the nucleus^26^,^58^,^59^. In our studies, treatment of Thiocolchicoside resulted in inhibition of ciliary Smo accumulation, as well as dispersion of a distinct subcellular localization of Smo from near the ciliary compartment to all over the cytoplasm by disrupting cytoplasmic MT (Figure S9). This suggests that MT dynamics are required for intracellular movement of Smo. It is also possible that Thiocolchicoside perturb MT-dependent recruitment of other Hh signaling components, such as Sufu and Gli, from the cytoplasm into cilia. Alternatively, it could influence the transport of Sufu and Gli into the nucleus, as observed for JK184, nocodazole and colchicine^26^,^58^,^59^, which is an independent mechanism of Hh regulation.

High levels of Hh target genes have been observed in both BCC and MB, which both require PC for tumorigenesis. Hh target gene activation induces ciliogenesis, which could be the reason for the high levels of Hh target genes observed in these cancer forms^34^,^36^,^37^. Aberrantly elevated Hh signaling is frequently found in PDAC, but PC function in cancer initiation and progression is far from being understood. Several studies have shown that Hh ligands are secreted from pancreatic cancer cells and their earliest premalignant lesions, known as pancreatic interepithelial neoplasia (PanIN)^60^,^61^. Thus, secreted Hh ligands stimulate the PC-dependent Hh signaling pathway in the surrounding stroma compartment, where PC are abundant, promoting the formation of the intense desmoplastic reaction associated with the disease^62^.

Oncogenic Kras-derived PDAC and PanIN lesions are devoid of PC^63^. In contrast, we demonstrated that PC can be found in pancreatic cancer cells of the KPC-SmoM2 transgenic mouse model (Fig. 4). We have identified this model to be similar to BCC and MB models as they all have active mutations of Hh signaling components, in addition to a high number of PC^7^,^37^. Moreover, using human pancreatic cancer cell lines and cancer cells from pancreatic ductal adenocarcinoma patients, Sakamoto and his colleagues showed the presence of PC in cancer cells, which is correlated with poor prognosis of the disease^64^. Thus, it is likely that PC-dependent Hh signaling promotes ciliogenesis as well as cancer initiation and progression and might therefore be a critical target for future pancreatic cancer therapies.

Hh signaling leads to increased cell proliferation and tumor formation. Inhibition of Hh signaling with a Smo antagonist, Cyclopamine, substantially blocks tumor formation and prolongs survival in a transgenic mouse model of pancreatic cancer^60^,^65^. Moreover, the orally bioavailable IPI-269609, a semisynthetic analogue of Cyclopamine, prevents tumor initiation and metastatic spread in orthotopic xenografts of pancreatic cancer cells^66^. We have shown that sole treatment of compounds antagonizing oncogenic mutant SmoM2 effectively inhibited Gli expression and pancreatic cancer cell proliferation *in vitro* (Fig. 4). Likewise, pharmacological blockade of Hh signaling with Smo antagonists are effective in treating and preventing pancreatic cancer^67^,^68^. Most of Smo antagonists identified in our screening were effective at inhibiting both ligand-dependent and independent Hh pathways, suggesting that these compounds might be universally applicable to cancers associated with constitutive Hh signaling.

In this respect it is interesting to note that clinical trials on combination chemotherapy revealed more beneficial results than single agent chemotherapy for patients with advanced cancer. Several studies have reported a promising overall survival advantage with the combination of Itraconazole, a common antifungal agent and a potent inhibitor of the Hh pathway, and other chemotherapy drugs such as Gemcitabine, a nucleoside analog for treating metastatic pancreatic cancer, ovarian cancer and breast cancer^69^,^70^. Thus, synergistic effects of our newly identified drugs through complementary yet independent mechanisms should be tested in *in vitro* and *in vivo* cancer systems for the treatment of the high number of Hh-related cancers.

## Materials and Methods

### Ethics statement

All mice were housed in the central facilities at HMGU or Technische Universität München in accordance with the German animal welfare legislation and acknowledged guidelines of the Society of Laboratory Animals (GV-SOLAS) and of the Federation of Laboratory Animal Science Associations (FELASA). All animal studies were approved by the Institutional Animal Care and Use Committees (IACUC) of HMGU or Technische Universität München, Regierung von Oberbayern and these studies were carried out in accordance with the approved guidelines. All scarification of mice at embryonic stages was not subject to regulatory authorization.

### Compounds

Compound 1–10, Bromopride (Prestw-704), Droperidol (Prestw-360), Sulpiride (Prestw-56), Tripelennamine hydrochloride (Prestw-1199), Cyclizine hydrochloride (Prestw-510), Palonosectron hydrochloride (Prestw-1783), (-)-MK801 hydrogen maleate (Prestw-935), Tramadol hydrochloride (Prestw-1465), Lorglumide sodium salt (Prestw-915) and Thiocolchicoside (Prestw-539) were purchased from Prestwick Chemical (Illkirch, France). Cyclopamine (Cyc, C4116) was obtained from Sigma, and Vismodegib (GDC-0499, S1082) was obtained from Selleck Chemicals. BODIPY-Cyclopamine (2160-50, 250) used for a BODIPY-Cyclopamine competition assay was purchased from Biovision.

### Generation of immortalized limb bud cells (LB) cells stably expressing RFP-tagged SmoM2 and Venus-tagged Arl13b for an image-based high content screening

Immortalized limb bud cells were generated from forelimbs of E12.5 wild-type mouse embryos according to the procedure of Jung *et al.* submitted.

We then generated an oncogenic point mutation in Smo (W535L) according to the manufacturer’s protocol of QuikChange site-directed mutagenesis kit (Agilent Technologies) with mutagenic oligonucleotide primers (SmoM2-For: 5′- GCC ATG AGC ACC CTC GTC TGG ACC AAG GCC AC -3′; SmoM2-Rev: 5′- GT GGC CTT GGT CCA GAC GAG GGT GCT CAT GGC -3′) and a Smo wild-type plasmid DNA template, pCAG-RFP-SmoWT-2A-Venus-Arl13b.

To generate stable populations of LB cells, either a linearized pCAG-RFP-SmoWT-2A-Venus-Arl13b or pCAG-RFP-SmoM2-2A-Venus-Arl13b were transfected into wild-type LB cells using TransFectin (Bio-Rad). After 48h post-transfection, the cells were selected with puromycin (1 μg/ml) for 2–4 weeks and all cells were pooled, expanded and maintained under puromycin selection. Ligand-independent Hh pathway activation in SmoM2 expressing LB cells, when compared to SmoWT expressing LB cells, was confirmed by immunostaining against RFP (1:1000, Chromotek) and GFP (1:1000, Aves Labs) antibodies, and by immunoblotting with Gli1 (1:1000, NEB) and Gli2 (1:1000, R&D system) antibodies.

### Screening instruments

Plate and liquid handling was performed using a HTS platform system composed of a Sciclone G3 Liquid Handler from PerkinElmer (Waltham, MA, USA) with a Mitsubishi robotic arm (Mitsubishi Electric, RV-3S11), a MultiFlo^TM^ Dispenser (Biotek Instruments, Bad Friedrichshall, Germany) and a Cytomat^TM^ Incubator (Thermo Fisher Scientific, Waltham, MA, USA). Cell seeding and assays were performed in black 384-well CellCarrier^TM^ plates (PerkinElmer, 6007558). The diverse small molecule library used in HTS was acquired from Prestwick Chemical (Illkirch, France). We tested 960 small molecule compounds of the Prestwick Chemical library, which are 100% approved drugs (Food and Drug Administration (FDA), European Medicines Agency (EMA) and other agencies). The purity of the compounds was >90% as reported by the provider of the compounds. Cells were seeded 40h before treatment in 384-well microplates with a cell number of 4 × 10^4^ cells/well. This resulted in about 80–90% confluent after 16 h and in a confluent layer after serum starvation for another 24 h (time of compound addition). Image acquisition and image-based quantification was performed using the Operetta^®^/Harmony^®^ high-throughput imaging platform (PerkinElmer, USA).

### High content screening assay

The LB cells stably expressing RFP tagged SmoM2 and Venus tagged Arl13b were cultured in standard DMEM medium (Invitrogen), supplemented with 10% FCS, 2 mM L-Glutamine, 1% Penicillin/Streptomycin and puromycin (1 μg/ml). For the screening, cells were washed with 1x PBS, trypsinized and resuspended in cell culture medium. The cell suspension (4 × 10^4^ cells in 50 μl per well) was dispensed into 384-well plates (PerkinElmer Cell carrier 384) and incubated (37 °C; 5% CO2) overnight. The next day, to induce ciliogenesis and ligand-independent ciliary SmoM2 accumulation, cells were serum deprived for 24 h and subsequently pretreated either with compound (1 mM stock solution) dissolved in 100% dimethyl sulfoxide (DMSO) or DMSO alone. 0.4 μl of compounds/DMSO were transferred to 50 μl DMEM medium, supplemented with 2 mM L-Glutamine and 1% Penicillin/Streptomycin per well to keep the final compound concentration at 8 μM and the DMSO volume concentration at 0.8%. The cells were then incubated (37 °C; 5% CO2) for 24 h prior to fixation and antibody staining.

Cells were fixed and permeabilized with 100% ice-cold methanol for 5min at −20 degree and subsequently treated with 10% neutral buffered formalin (Sigma) for 5min at room temperature. After blocking (10% donkey serum, 1% BSA and 5% FCS, 0.5% Tween-20 in PBS) for 2 h, cells were incubated with primary antibodies, GFP (1:1000, Aves Labs) and RFP (1:1000, Chromotek), overnight at 4 °C. The following secondary antibodies were applied for 2 h at room temperature: anti-chicken IgY-Cy2 (1:800, Dianova) and anti-rat IgG-Cy3 (1:800, Dianova). After DAPI staining (50 ng/ml), plates were recorded using the automated Operetta^®^ microscope with the 40 x high NA objective for high-resolution images (PerkinElmer, USA).

To identify drugs selectively disrupting SmoM2 trafficking to the PC without affecting ciliogenesis, 3 fields with 3 planes each (9 images in total) were taken randomly for each treatment. This resulted in a cell number of approximately 100 cells of each condition in control wells with DMSO. The acquired images were then analyzed with the Harmony^®^ software (PerkinElmer, USA) that was based on counting nuclei (Hoechst), followed by texture analysis (Venus channel). In more detail, the determination of cell count, nuclear intensity and area was based on the detection of Hoechst stained nuclei. Cells with a size smaller than 100 μm^2^ and with high nuclear intensity were not considered for cell count and thus excluded. In the next step, the complete images were searched for typical cilia texture regions. Using this method, we have combined image texture-based analysis to define cilia structures (Venus-tagged Arl13b positive structures) with quantitative co-localization analysis of RFP-tagged SmoM2 signal. We were able to calculate the mean cilia number per cell and to identify cilia with abrogated SmoM2 intensity. In addition, we excluded cytotoxic small molecules that resulted in less than 50 selected cells per well. With this strategy we initially identified 96 non-toxic primary hits. For all the selected hits the acquired images were reanalyzed visually to assure correct data analysis. Thereby we reduced the hit rate to 36 small molecules.

### Ciliary localization of endogenous Smo and Hh target gene activation in NIH3T3 cells, Ptch1^−/−^ MEFs, and primary PDAC cells

NIH3T3 cells, Ptch1^−/−^ MEFs (a gift from Prof. Rune Toftgård, Karolinska Institute, Sweden)^71^, and primary PDAC cells were maintained in standard DMEM medium (Invitrogen), supplemented with 10% FCS, 2 mM L-Glutamine and 1% Penicillin/Streptomycin. Cells were seeded at approximately 70–80% confluence. The following day, NIH3T3 cells were subjected to serum deprivation overnight to induce ciliogenesis and subsequently treated with Shh (500 ng/ml, R&D systems) plus compounds, Cyc, or DMSO with indicated concentration in figures or figure legends for an additional 24 h. For compounds treatment in Ptch1^−/−^ MEFs, confluent cells were subject to serum starvation with treatment of compounds, Cyc, or DMSO for 24 h. To check the compounds effect on Primary PDAC cells, when cells reached confluence in the maintaining medium, compounds, Cyc, or DMSO were added to the cells for 24 h.

### Isolation of primary cancer cells from PDAC mouse models

The murine primary dispersed pancreatic tumor cells (Figs 4, 5 and Figures S10, S11, S12) were isolated from murine pancreatic cancer as described^72^. In brief, tumors were digested in 10 mL Dulbecco’s modified Eagle medium (DMEM) (Sigma) containing 150 U/mL collagenase Type 2 (Worthington). Digested tumors were dispersed into single cell suspension, resuspended in DMEM medium containing 10% FCS and cultured in a 37 °C, 5% CO2, humidified incubator. The KPC mouse strain was described recently^49^,^50^. The *LSL-Rosa26*^*SmoM2−YFP/*^+ mouse strain was obtained from Jackson Laboratories (Jackson lab, strain number: 005130)^51^. Identity of the murine primary pancreatic cancer cells was verified using genotyping PCR.

### 3D spheroid culture of primary cancer cells from PDAC mouse models

Primary PDAC cells were cultured in standard DMEM medium (Invitrogen), supplemented with 10% FCS, 2 mM L-Glutamine and 1% Penicillin/Streptomycin. For 3D Matrigel-embedded culture, 100 cells were suspended in 10 μl of complete culture medium, gently mixed with 50ul of growth factor-reduced Matrigel (BD Biosciences) and placed into a 96 well imaging glass bottom microplate (PAA). After polymerization of Matrigel at 37 °C incubator for 15min, 100 μl of the culture medium was added on top of the 3D Matrigel and cells were grown for 5–7 days, changing medium every 2–3 days. To investigate the effect of compounds on 3D tumor spheroids, the spheroids were incubated with the culturing medium containing 10 μM of compound and the medium was then changed every 24 h for 3 days.

### BODIPY-Cyclopamine competition assay

HEK293T cells were plated at approximately 70% confluence. The following day, the cells were transiently transfected with a Smo construct, pCS107-SmoWT-Myc^14^, using PEI (polyethylenimine, Polysciences) as previously described^73^. 24 h after transfection, the cells were incubated with 20 nM BODIPY-Cyclopamine and 10 μM Cyc or 10 μM compounds for 4 h. The cells were then trypsinized, centrifuged, and resuspended in PBS, and BODIPY fluorescence signal were detected with the FL1 channel (505–530 nm) of FACS machine (FACSAria, BD). FACS data was carefully analyzed with FlowJo software.

### GBS-GFP fluorescence intensity measurement

GBS-GFP MEFs were generated from E12.5 transgenic reporter mouse-Tg (GBS-GFP) embryos^40^. Cells were seeded at approximately 70–80% confluence. The following day, cells were subjected to serum deprivation and subsequently treated with Shh (500 ng/ml, R&D systems) plus compounds, Cyc, or DMSO for 24 h. Cells were fixed with 4% PFA for 10 min and subsequently stained with an antibody against GFP (1:500, Biotrend). 10 images of GFP staining per condition (in total, over 1000 cells) were randomly taken using a Leica laser-scanning SP5 confocal microscope with a 63x objective. Quantitative analysis of fluorescence intensities was carried out using Imaris software.

### Production of full-length hGprasp2

A construct expressing the full length hGPRASP2 was transformed into E. coli Rosetta2 (DE3) and cultured at 20 °C in ZYM 5052 auto-induction medium^74^. After harvesting the cells were lysed by sonication and the His6-tagged protein purified by immobilized metal affinity chromatography (IMAC) using a 5 mL HiTrap Chelating HP column (GE Healthcare). The eluted protein was dialyzed overnight in the presence of TEV protease and further purified by a second IMAC step. Next it was applied to size exclusion chromatography using a HiLoad 16/600 Superdex 200 column (GE Healthcare) and fractions containing full length hGPRASP2 were pooled and concentrated to approx. 5 mg/mL and stored at 4 °C. The protein concentration was determined by measuring the absorbance at 280 nm using a specific absorbance of 1.170 ml/mg.

### NMR spectroscopy

NMR experiments were recorded on a 600 MHz spectrometer equipped with a CPQCI probehead at 298 K using hGPRASP2 and peptide (hSMO-WT, aa539–552; hSMO-CLD, aa539–552; hSMO-WT, aa533–552; hSMOM2, aa533–552-W535L, PSL GmbH) solutions in 50 mM sodium phosphate pH 8, 50 mM NaCl, 0.002% NaN3, 100 μM DL-Dithiothreitol (90% H_2_O/10% D_2_O). Interaction studies between hGPRASP2 and hSMO peptides were performed using 100 μM peptide by recording 1D proton experiments before and after addition of hGPRASP2 to a final concentration of 0.78 μM. Compound interference of the hGPRASP2-hSMO interaction was performed using 100 μM hSMOM2 by recording 1D proton experiments before and after addition of hGPRASP2 previously incubated with compounds (or DMSO-d6) to a final concentration of 0.78 μM hGPRASP2 and 78 μM compounds. 1D proton experiments were performed using a WATERGATE pulse sequence with 32k time domain points and 256 scans.

### Immunoblotting

RIPA buffer with protease inhibitor (Sigma) was used for preparing protein lysates. Immunoblottings were performed following a standard protocol with indicated antibodies: Gli1 (1:1000, NEB), Gli2 (1:1000, R&D System), Gprasp2 (1:1000, kindly provided by Dr. Erich Wanker, Max Planck Institute, Germany), CyclinD1 (1:1000, NEB) Smo (1:1000, Abcam), GFP (1:1000, Biotrend), α-Tubulin (1:5000, Sigma), GAPDH (1:6000, Merck Bioscience) and Actin (1:5000, BD). Protein bands were visualized by chemiluminescence (ECL, Millipore) and subsequently exposed to Hyperfilms (GE healthcare; Agfa healthcare). NIH ImageJ software was used for immunoblot quantification.

### Immunofluorescence staining

For immunocytochemistry, cells were fixed and permeabilized with 100% ice-cold methanol for 5min at −20 degree and subsequently treated with 10% neutral buffered formalin (Sigma) for 5min at room temperature.

For immunohistochemistry on paraffin sections, tissue specimens were first fixed in 4% buffered formalin, embedded in paraffin and sectioned (3 μm thick). After deparaffinization through Xylene and EtOH series steps, the tissue sections were washed with PBS and preceded to antigen-retrieval with antigen unmasking solution (Vector Lab. Inc.).

After blocking (10% donkey serum, 1% BSA and 5% FCS, 0.5% Tween-20 in PBS) for 2 h, the indicated concentration of primary antibodies was applied overnight at 4 °C: α-tub (1:1000, Sigma), Ace-tub (1:1000, Sigma), Smo (1:1000, Abcam), Gprasp2 (1:100, kindly provided by Dr. Erich Wanker, Max Planck Institute, Germany), GFP (1:500, Biotrend), GFP (1:1000, Aves Labs), RFP (1:1000, Chromotek) and Pericentrin (1:500, Covance). The following secondary antibodies were treated for 2 h at room temperature: anti-mouse IgG-649 (1:800, Invitrogen), anti-rabbit IgG-555 (1:800, Invitrogen), anti-rabbit IgG-488 (1:800, Invitrogen), anti-chicken IgY-Cy2 (1:800, Dianova) and anti-rat IgG-Cy3 (1:800, Dianova).

After staining with DAPI (50 ng/ml), the samples were mounted with ProLong gold antifade reagent (Invitrogen) and images were acquired by using a Leica laser-scanning SP5 confocal microscope with a 63x objective. Quantitative analysis of fluorescence intensities and 3D visualization and reconstruction of Z-stack images of the spheroids were performed with Imaris software.

### BrdU cell proliferation assay

Primary PDAC cells were plated onto 96 well plates at approximately 60–70% confluence and the following day, cells in the maintaining medium (10% FCS, 2 mM L-Glutamine and 1% Penicillin/Streptomycin) were treated with compounds, Cyc, or DMSO for 24 h. Cells were subsequently incubated with BrdU for 3 h and, according to the manufacturer’s protocol of BrdU cell proliferation assay kit (Cell Signaling), the assay was performed. Absorbance was read using a spectrophotometric microplate reader (Bio-Rad) set at a single wavelength at 450 nm.

The IC50 values for primary PDAC cell proliferation was determined by statistical and dose-response curve analyses using using GraphPrad Prism software.

### Cell cycle analysis by Propidium Iodide (PI)

Primary PDAC cells were plated onto 6 well plates at approximately 60–70% confluence and the following day, cells in the maintaining medium (10% FCS, 2 mM L-Glutamine and 1% Penicillin/Streptomycin) were treated with compounds, Cyc, or DMSO for 24 h. Cells were then trypsinized, centrifuged and resuspended in PBS. For fixation, 100% EtOH was added to the resuspended cell pellet at a final concentration of 70% and incubated on ice for 15min. Cells were then centrifuged again, resuspended in PI staining solution (50 μg/ml PI, Sigma, 0.1 mg/ml RNase A, 0.05% Triton X-100) and incubated for 40 min at 37 °C. After PI staining, cells were finally resuspened in PBS and PI signal was detected in the FL-2 channel (561 nm) on a FACS machine (FACSAria, BD). Cell cycle analysis was carried out with FlowJo software.

## Additional Information

**How to cite this article**: Jung, B. *et al.* Novel small molecules targeting ciliary transport of Smoothened and oncogenic Hedgehog pathway activation. *Sci. Rep.* **6**, 22540; doi: 10.1038/srep22540 (2016).

## Supplementary Material

Supplementary Information

Supplementary Movie 1

Supplementary Movie 2
