# Supplementary material for: Novel small molecules targeting ciliary transport of Smoothened and oncogenic Hedgehog pathway activation
Source: Sci Rep. 2016 Mar 2;6:22540. doi: 10.1038/srep22540 (PMC4773810; doi:10.1038/srep22540)
Supplement: Supplementary Information [file srep22540-s1.pdf]

Supplementary Information

Novel small molecules targeting ciliary transport of Smoothened and oncogenic Hedgehog pathway activation

by

Bomi Jung[1,2], Ana C. Messias[3,5], Kenji Schorpp[4], Arie Geerlof[3], Günter Schneider[6,7], Dieter Saur[6,7,9,10], Kamyar Hadian[4], Michael Sattler[3,5], Erich E. Wanker[11], Stefan Hasenöder[1,2] & Heiko Lickert[1,2,7,8]

[1]Institute of Diabetes and Regeneration Research, [2]Institute of Stem Cell Research, [3]Institute of Structural Biology, [4]Assay Development and Screening Platform, Helmholtz Zentrum München, Germany,
[5]Center for Integrated Protein Science Munich at Biomolecular NMR Spectroscopy, Department Chemistry, Technische Universität München, 85747 Garching, Germany
[6]Department of Internal Medicine II, Klinikum rechts der Isar, [7]Technische Universität München, München, Germany. [8]German Center for Diabetes Research (DZD), Germany. [9]German Cancer Consortium (DKTK), Heidelberg, Germany. [10]German Cancer Research Center (DKFZ), Heidelberg, Germany. [11]Neuroproteomics, Max Delbrueck Center for Molecular Medicine, 13125 Berlin, Germany

Correspondence and requests for materials should be addressed to H.L. (heiko.lickert@helmholtz-muenchen.de)

Heiko Lickert
Helmholtz Zentrum München
Parkring11
D-85748, Garching
Germany
PHONE: +49 89 3187 3760
FAX: +49 89 3187 3761

Supplementary Materials and Methods

Cell viability assay

Cells were seeded at approximately 70-80% confluence on 96 well plates. The following day, cells were subjected to serum deprivation and subsequently treated with Shh (500 ng/ml, R&D systems) plus one of the compounds or DMSO for 24 or 48 h. AlamarBlue reagent (Invitrogen) was directly added to cells in the culture medium according to the manufacturer's protocol and incubated for 3 h at 37 degree in a cell culture incubator. Absorbance of alamarBlue was monitored at 570nm.

Quantitative PCR

Total RNA was obtained using Trizol and miRNeasy micro kit (Qiagen) according to the manufacturer's protocol. Subsequently, cDNA was prepared with the Super Script Synthesis System (Invitrogen). TaqMan® qPCR was performed by using 25 ng of cDNA per qPCR reaction and the following probes according to the manufacturing instruction; TaqMan probes: Gli1 (Mm00494654_m1), Ptch1 (Mm00436026_m1), GFP (Mr04329676_mr), GAPDH (Mm99999915_g1). The analysis was carried out in biological triplicates.

Hh/Gli1 target gene activation in Sufu$^{-/-}$ MEFs

Sufu$^{-/-}$ MEFs (a gift from Prof. Rune Toftgård, Karolinska Institute, Sweden) were maintained in standard DMEM medium (Invitrogen), supplemented with 10% FCS, 2 mM L-Glutamine and 1% Penicillin/Streptomycin. Cells were seeded at approximately 70-80% confluence. The following day, Sufu$^{-/-}$ MEFs were concurrently subjected to serum deprivation and the treatment of compounds, Cyc, or DMSO for 24 h. To check the effect of compounds on Sufu$^{-/-}$ MEFs, the total RNA was isolated and cDNA was prepared as described above. Gli1 mRNA expression levels were determined by quantitative PCR using TaqMan Gli1 probe as mentioned above.

Immunoprecipitation

HEK293T cells were transiently transfected using PEI (polyethylenimine, Polysciences) as previously described[1]. Importantly, the cells were treated with 20uM compounds immediately after transfection to prevent protein-protein interactions. 24h after transfection with compound treatment, the cells were lysed in IP lysis buffer (30 mM Tris-HCl, pH 7.5, 150 mM NaCl, 0.5% NP-40) containing protease inhibitor cocktail (Roche) and after centrifugation, the supernatant was subsequently incubated with

Strep-Tactin superflow resin (IBA) for overnight at 4°C on a rotating wheel (14rpm). After extensive washing with IP lysis buffer, the protein complexes were analyzed by immunoblotting with anti-HA primary antibody (1:1000, Sigma).

Compound	Target	UniProt ID	WR Motif
	Smoothened	Q99835	aa 539- ATLLIWRRTWCR*
1, 2, 3	Dopamine D2 receptor	P14416	aa 429- FNIEFRKAFLKILHC*
3	Dopamine D3 receptor	P35462	aa 388- FNIEFRKAFLKILSC*
4, 5	Histamine H1 receptor	P35367	aa 473- ENFKKTFKRILH*
6	Serotonin 3A receptor	P46098	aa 340- PAWLRHLVLERIAWLLCL*<br>aa 440- RDWLRVGSVL*
7	NMDA1 receptor	Q05586	aa 834- EIAYKRH#<br>aa 857- VWRKNLQD#
7	NMDA 2A receptor	Q12879	aa 839- HLFYWKLRFCFT#<br>aa1011- RQLWKKSV#
8	Mu-type opioid receptor	P35372	aa343- ENFKRCFREF*
9	Cholecystokinin receptor A	P32238	aa375- KRFRLGFMAT#
10	$GABA_A$ receptor subunit alpha1	P14867	aa335- NYFTKRGYAWD#
10	$GABA_A$ receptor subunit gamma2	P18507	aa429- WRHGRIHIR#

Supplementary Table 1. Targets of compounds 1-10 and their C-terminus conserved hydrophobic and basic residue motifs (F/WR/K).

Comparison of the motif (F/WR/K) in helix VIII of GPCRs or in predicted cytoplasmic regions of target receptors. Amino acid sequences highlighted in yellow are either helices seen in structures deposited at the PDB (*) or predicted by HNN software (#).

Supplementary Figure legends

Supplementary Figure 1. Demonstration of ligand-independent Hh pathway activation in SmoM2 expressing LB cells.

(A) Experimental scheme to generate SmoM2 expressing LB cells for high content screening. (B) Representative images of CLSM and (C) quantification of ciliary Smo in LB cells treated with or without Shh. Smo and Arl13b were stained with antibodies to RFP and Venus respectively. (D) The expression levels of Gli1, Gli2, RFP-tagged Smo protein in LB cells stably expressing RFP-tagged SmoWT or SmoM2 and Venus-tagged Arl13b after treatment with or without Shh. Graphs indicate quantification of immunoblot data that show the mean fold change of protein normalized to α-Tub levels.

Scale bar = 25 μm. > 100 cilia were analyzed per condition. All error bars represent the mean SD of three independent experiments. A two tailed unpaired *t-test* is used for statistical data analysis. (* = *p < 0.1, ** = p < 0.01, # = p < 0.001*)

Supplementary Figure 2. High content screening for identification of small molecule inhibitors of ciliary accumulation of oncogenic SmoM2.

(A) Experimental scheme for high content screening. (B) Representative high content screening images of LB cells stably expressing RFP-tagged SmoM2 and Venus-tagged Arl13b. SmoM2 and Arl13b were stained with antibodies to RFP and Venus respectively. (C) Quantification of fluorescence intensity of ciliary SmoM2 after hit validation.

Supplementary Figure 3. Compounds effect on ciliary SmoM2 localization in SmoM2 expressing LB cells.

Representative CLSM images of SmoM2 expressing LB cells treated with compound 1-10, Cyc, or GDC-0449. SmoM2 and Arl13b were visualized by staining with RFP and Venus antibodies respectively. Scale bar = 10 μm. > 100 cilia were analyzed per condition. All experiments were repeated at least three times.

Supplementary Figure 4. Compounds suppress ciliary accumulation of endogenous Smo.

Representative CLSM images of (A) NIH3T3 cells and (B) Ptch1$^{-/-}$ MEFs treated with compound 1-10, or Cyc. PC were stained with an antibody to Ace-Tub and Smo were visualized with a Smo antibody. Scale bar = 10 μm. > 100 cilia were analyzed per condition. All experiments were repeated at least three times.

Supplementary Figure 5. Cell viability is not affected by compounds treatment.

Cell viability measured after 24 or 48 h of exposure to various concentrations of compound 1-10.

All error bars represent the mean SD of three independent experiments. D = DMSO, 0.1%

Supplementary Figure 6. Determination of IC50 values for Hh pathway in GBS-GFP MEFs.

IC50 value determined by GFP and Ptch1 mRNA expression levels after treatment with various concentrations of compounds on GBS-GFP MEFs. Relative expression was normalized against GAPDH expression level. The data represent the mean values of three independent experiments.

Supplementary Figure 7. Compounds effect on Hh/Gli1 target gene expression in Sufu$^{-/-}$ MEFs.

Quantitative levels of Gli1 mRNA expression in Sufu$^{-/-}$ MEFs were measured by qPCR after treatment with compounds. Relative expression was normalized against GAPDH expression level.

All error bars represent the mean SD of three independent experiments. D = DMSO, 0.1%; Cyc, 10 µM; compound 1-4, 10 µM; compound 5-10, 20 µM

Supplementary Figure 8. All compounds, with the exception of compound 10, interfere with Gprasp2 binding to SmoWT and to SmoM2.

(A, B) Confirmation of compounds interference with Smo-Gprasp2 complex formation in co-immunoprecipitation (co-IP). HEK293T cells were transiently co-transfected with the indicated expression plasmids. Concurrently, the cells were treated with 20 µM compounds and 24 h later the cell lysates were subsequently subjected to immunoprecipitation. Note that 10% input and Smo-Gprasp2 protein interactions were detected by immunoblotting with the indicated antibodies.

Supplementary Figure 9. Ciliogenesis is unaffected by all compounds, but cytoplasmic distribution of Smo is disrupted by compound 10.

(A, E) Representative CLSM images of NIH3T3 cells treated with or without compound 1-10. (A) PC were stained with an antibody to Ace-Tub and basal bodies were visualized with a pericentrin antibody. (E) Microtubule network and endogenous Smo distribution were visualized with antibodies to α-Tub and Smo respectively. The quantification of (B) ciliation and cilia abnormality, (C) cilia length, and (D) centriole disengagement of NIH3T3 cells after treatment with compound 1-10.

Scale bar = 10 µm (A); 25 µm (E). > 100 cilia were analyzed per condition. All error bars represent the mean SD of three independent experiments. A two tailed unpaired *t-test* (B, D) and a one-way Anova (C) were used for statistical data analysis. (*# = p < 0.0001*)

Supplementary Figure 10. Compounds inhibit ciliary SmoM2 accumulation in primary SmoM2 PDAC cells.

Representative CLSM images of primary KPC-SmoM2 cells treated with compound 1-10 or Cyc. SmoM2 and Gprasp2 are visualized with antibodies to GFP and Gprasp2 respectively and PC are visualized with an antibody against Ace-Tub.

Scale bar = 10 µm. > 100 cilia were analyzed per condition. All experiments were repeated at least three times.

Supplementary Figure 11. Compounds do not effect on PDAC which do not show ciliary Smo-dependent Hh activity.

Representative CLSM images of (A, Movie S2) tissue specimen and (B) primary PDAC cells from a *Pdx1-Cre; LSL-Kras*$^{G12D/+}$*; LSL-Trp53*$^{R172H/+}$ (KPC) mouse strain. (A, B) Immunostaining with GFP antibody demonstrates specificity of the GFP signals shown in Figure 4 (A, B, F) and Figure 5 (B). Immunostaining against Ace-Tub and Smo antibodies show rare ciliation and no signs of Smo ciliary translocation. (C) Protein expression levels of Gli1, Gli2, (** indicates non-specific bands) and CyclinD1 after treatment with compound 1-10 on primary KPC cells. (D) Statistical analysis of BrdU incorporation after compound 1-10 treatment on primary KPC cells.

Scale bar = 25 µm. > 100 cilia were analyzed per condition. All error bars represent the mean SD of three independent experiments. D = DMSO, 0.1%; Cyc, 10 µM; compound 1-4, 10 µM; compound 5-10, 20 µM

Supplemental Figure 12. Effective concentration and half maximal inhibitory concentration (IC50) of compound 6 and 9 in Gli protein expression and PDAC tumor cell proliferation.

(A, B) The expression levels of Gli1 and Gli2 protein in primary Pan *KPC-SmoM2* cells after treatment with various concentrations of compound 6 or 9. Graphs indicate quantification of immunoblot data that show the mean fold change of protein normalized to α-Tub levels. (C, D) IC 50

value determined by BrdU incorporation assay after treatment with various concentrations of compound 6 or 9 on primary KPC-SmoM2 cells.

All error bars represent the mean SD of three independent experiments. A two tailed unpaired *t-test* is used for statistical data analysis. (* = $p < 0.01$, # = $p < 0.001$)

Supplemental Movie 1. CLSM images of tissue specimen from a *Pdx1-Cre; LSL-Kras*$^{G12D/+}$*; LSL-Trp53*$^{R172H/+}$*; LSL-Rosa26*$^{SmoM2-YFP/+}$ mouse strain. YFP-tagged SmoM2 is visualized with GFP antibody.

Supplemental Movie 2. CLSM images of tissue specimen from a *Pdx1-Cre; LSL-Kras*$^{G12D/+}$*; LSL-Trp53*$^{R172H/+}$ mouse strain. Immunostaining with GFP antibody demonstrates specificity of the GFP signals.

Supplemental References

1. Boussif, O. et al. A versatile vector for gene and oligonucleotide transfer into cells in culture and in vivo: Polyethylenimine. *Proc Natl Acad Sci U S A* **92**, 7297-7301 (1995).

Figure S1.

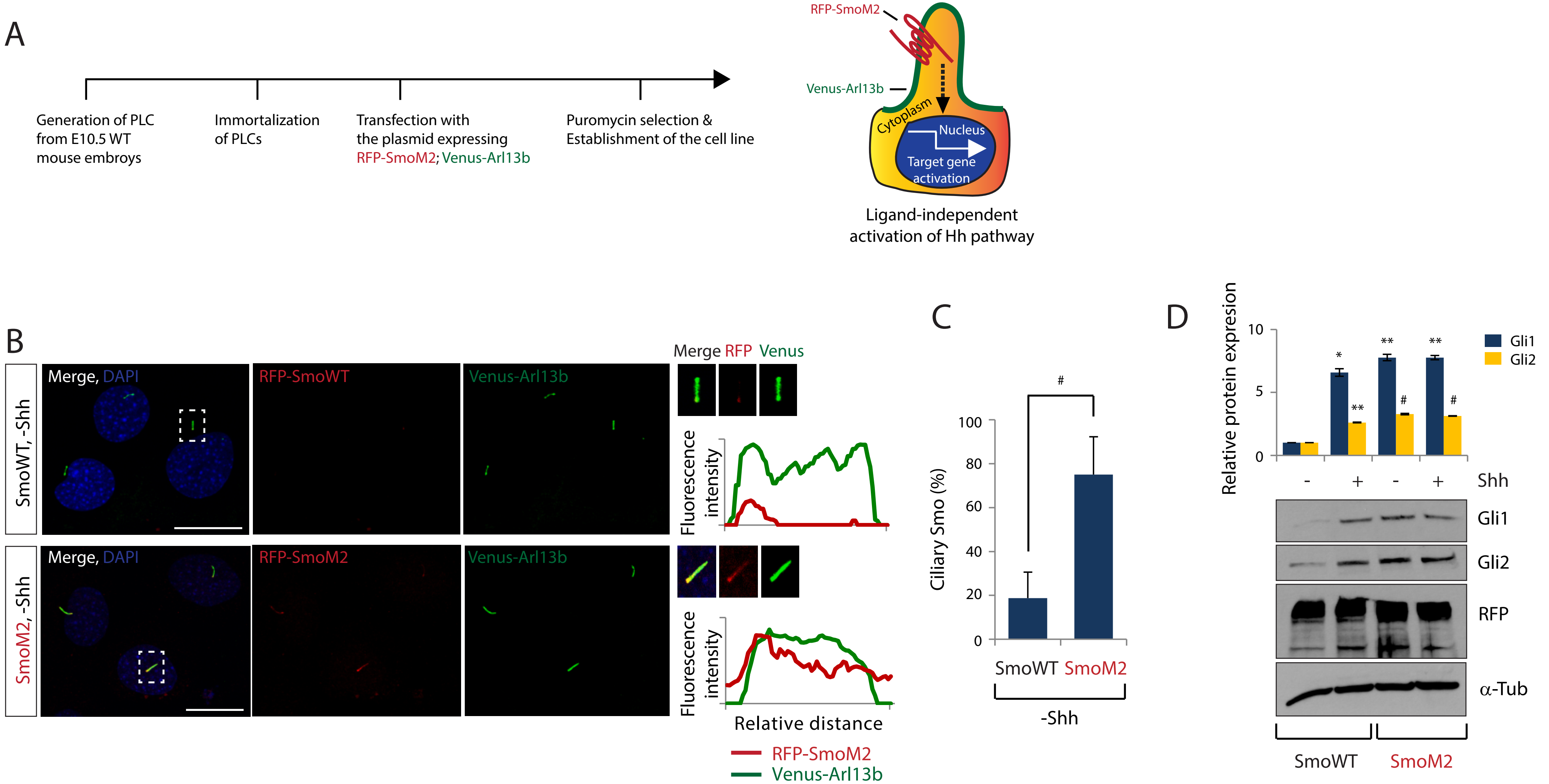

A
Generation of PLC from E10.5 WT mouse embroys
Immortalization of PLCs
Transfection with the plasmid expressing RFP-SmoM2; Venus-Arl13b
Puromycin selection & Establishment of the cell line
Ciliary SmoM2
RFP-SmoM2
Venus-Arl13b
Cytoplasm
Nucleus
Target gene activation
Ligand-independent activation of Hh pathway
B
SmoWT, -Shh
SmoM2, -Shh
Merge, DAPI
RFP-SmoWT
RFP-SmoM2
Venus-Arl13b
Merge RFP Venus
Fluorescence intensity
Relative distance
RFP-SmoM2
Venus-Arl13b
C
Ciliary Smo (%)
#
0
20
40
60
80
100
SmoWT SmoM2
-Shh
D
Relative protein expresion
10
5
0
Gli1
Gli2
*
**
**
**
#
#
- + - +
Shh
Gli1
Gli2
RFP
α-Tub
SmoWT
SmoM2

Figure S2.

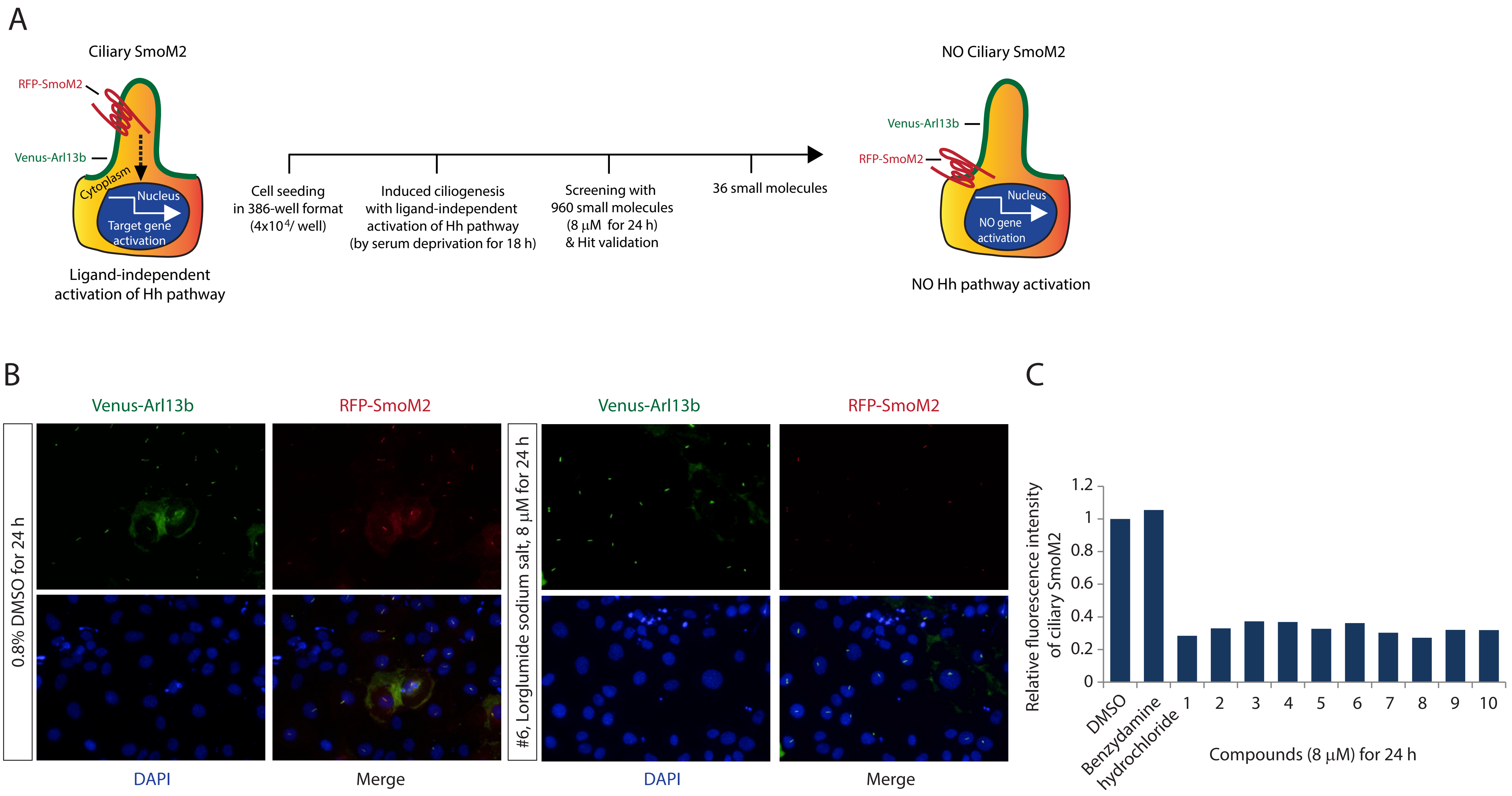
A
Ciliary SmoM2
RFP-SmoM2
Venus-Arl13b
Cytoplasm
Nucleus
Target gene activation
Ligand-independent activation of Hh pathway
Cell seeding in 386-well format (4x10^4/ well)
Induced ciliogenesis with ligand-independent activation of Hh pathway (by serum deprivation for 18 h)
Screening with 960 small molecules (8 μM for 24 h) & Hit validation
36 small molecules
NO Ciliary SmoM2
Venus-Arl13b
RFP-SmoM2
Nucleus
NO gene activation
NO Hh pathway activation
B
Venus-Arl13b
RFP-SmoM2
0.8% DMSO for 24 h
DAPI
Merge
#6, Lorglumide sodium salt, 8 μM for 24 h
C
Relative fluorescence intensity of ciliary SmoM2
1.2
1
0.8
0.6
0.4
0.2
0
DMSO
Benzydamine hydrochloride
1
2
3
4
5
6
7
8
9
10
Compounds (8 μM) for 24 h

Figure S3.

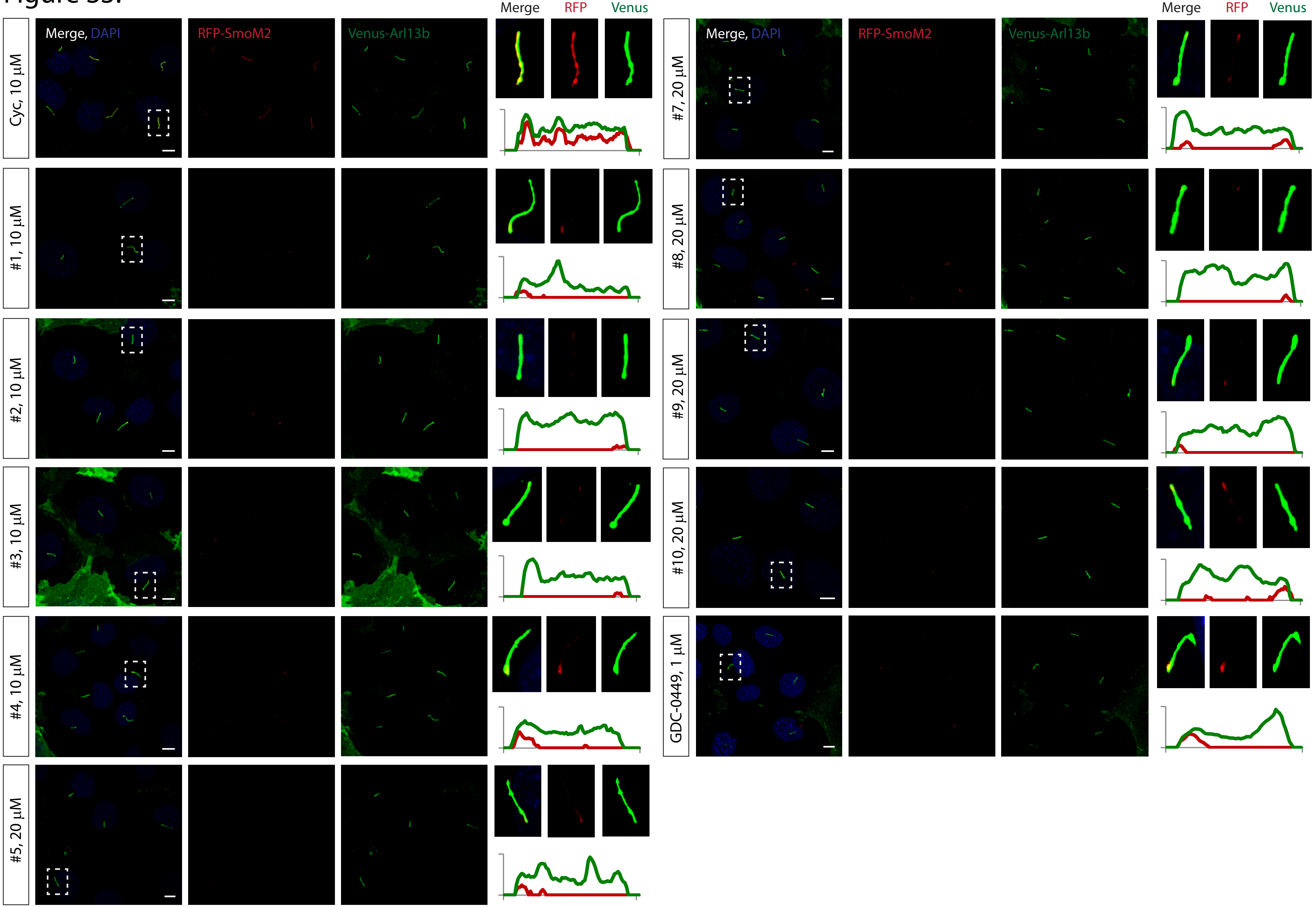

Merge
RFP
Venus
Merge, DAPI
RFP-SmoM2
Venus-Arl13b
Cyc, 10 µM
#1, 10 µM
#2, 10 µM
#3, 10 µM
#4, 10 µM
#5, 20 µM
#7, 20 µM
#8, 20 µM
#9, 20 µM
#10, 20 µM
GDC-0449, 1 µM

Figure S4.

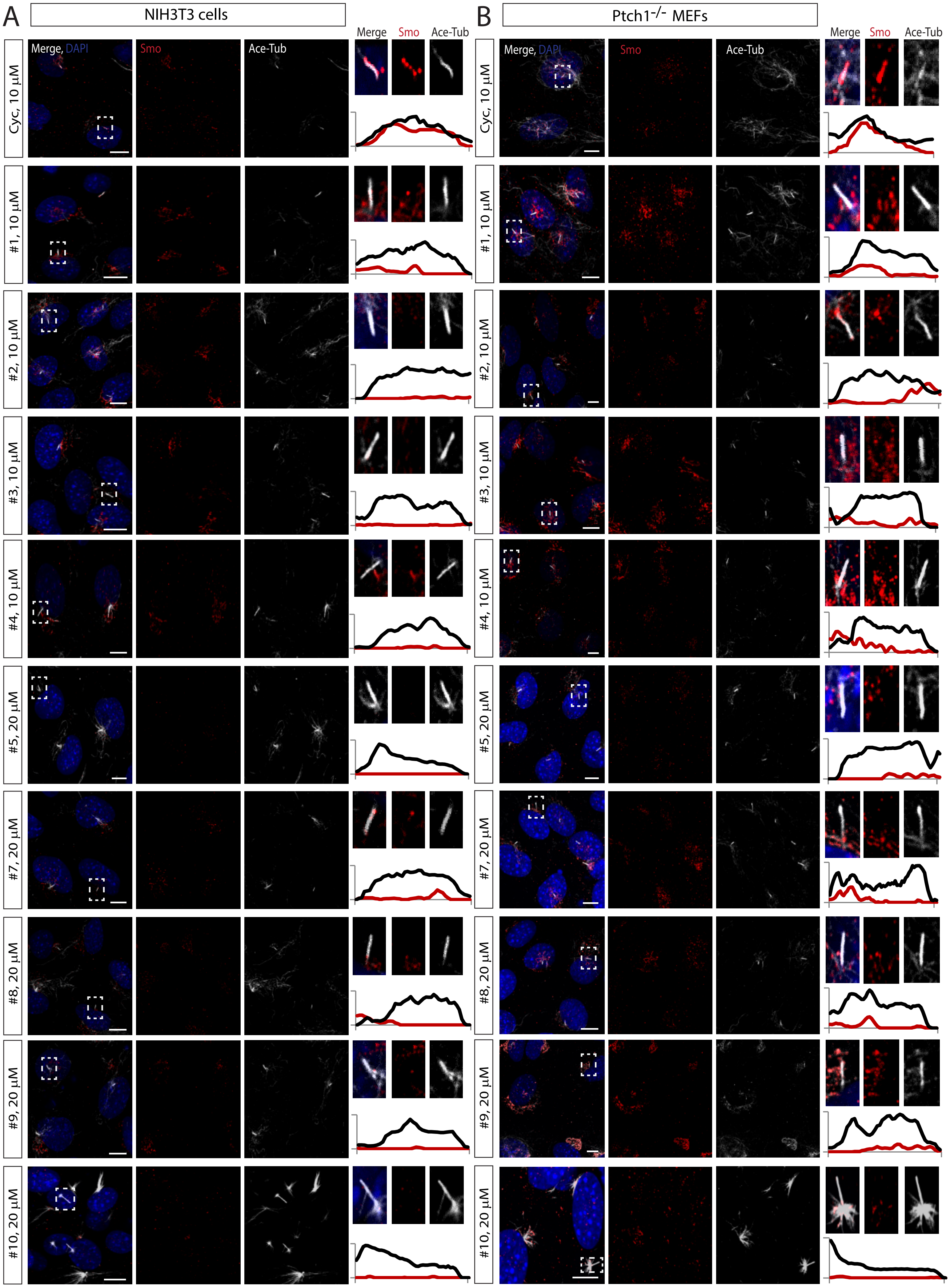
A
NIH3T3 cells
B
Ptch1-/- MEFs
Merge, DAPI
Smo
Ace-Tub
Merge
Smo
Ace-Tub
Cyc, 10 µM
#1, 10 µM
#2, 10 µM
#3, 10 µM
#4, 10 µM
#5, 20 µM
#7, 20 µM
#8, 20 µM
#9, 20 µM
#10, 20 µM

Figure S5.

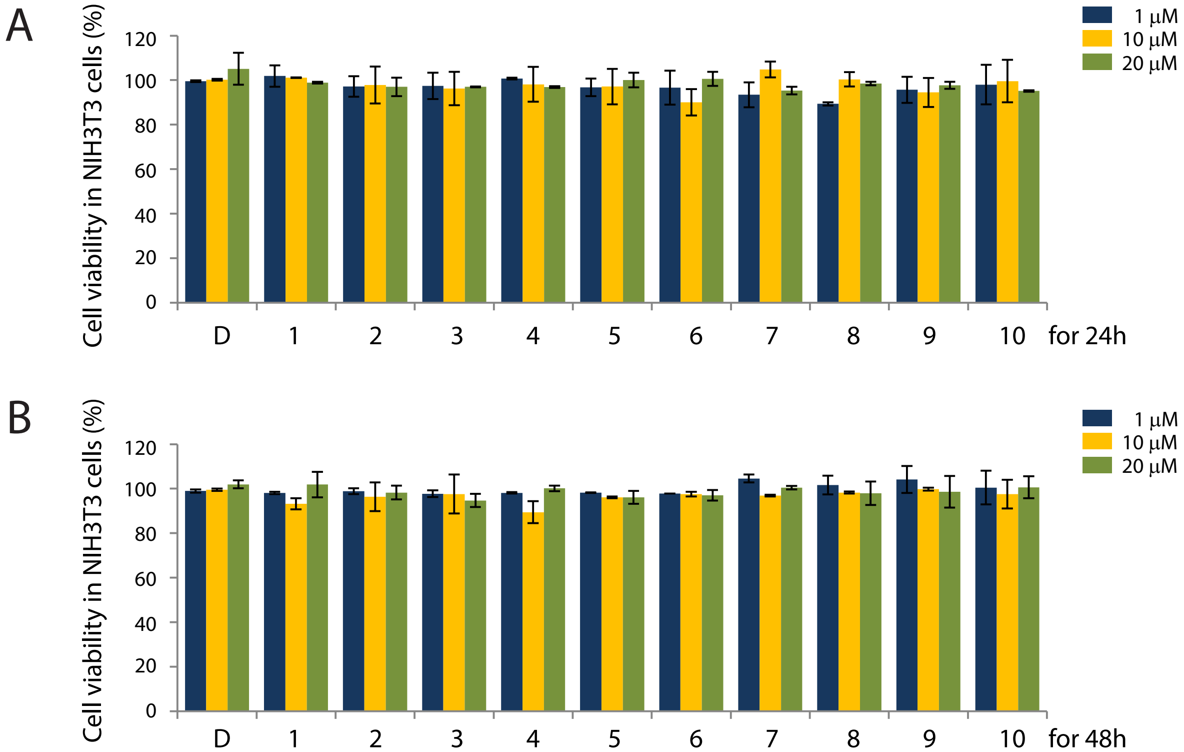

A
Cell viability in NIH3T3 cells (%)
120
100
80
60
40
20
0
D
1
2
3
4
5
6
7
8
9
10
for 24h
1 μM
10 μM
20 μM
B
Cell viability in NIH3T3 cells (%)
120
100
80
60
40
20
0
D
1
2
3
4
5
6
7
8
9
10
for 48h
1 μM
10 μM
20 μM

Figure S6.

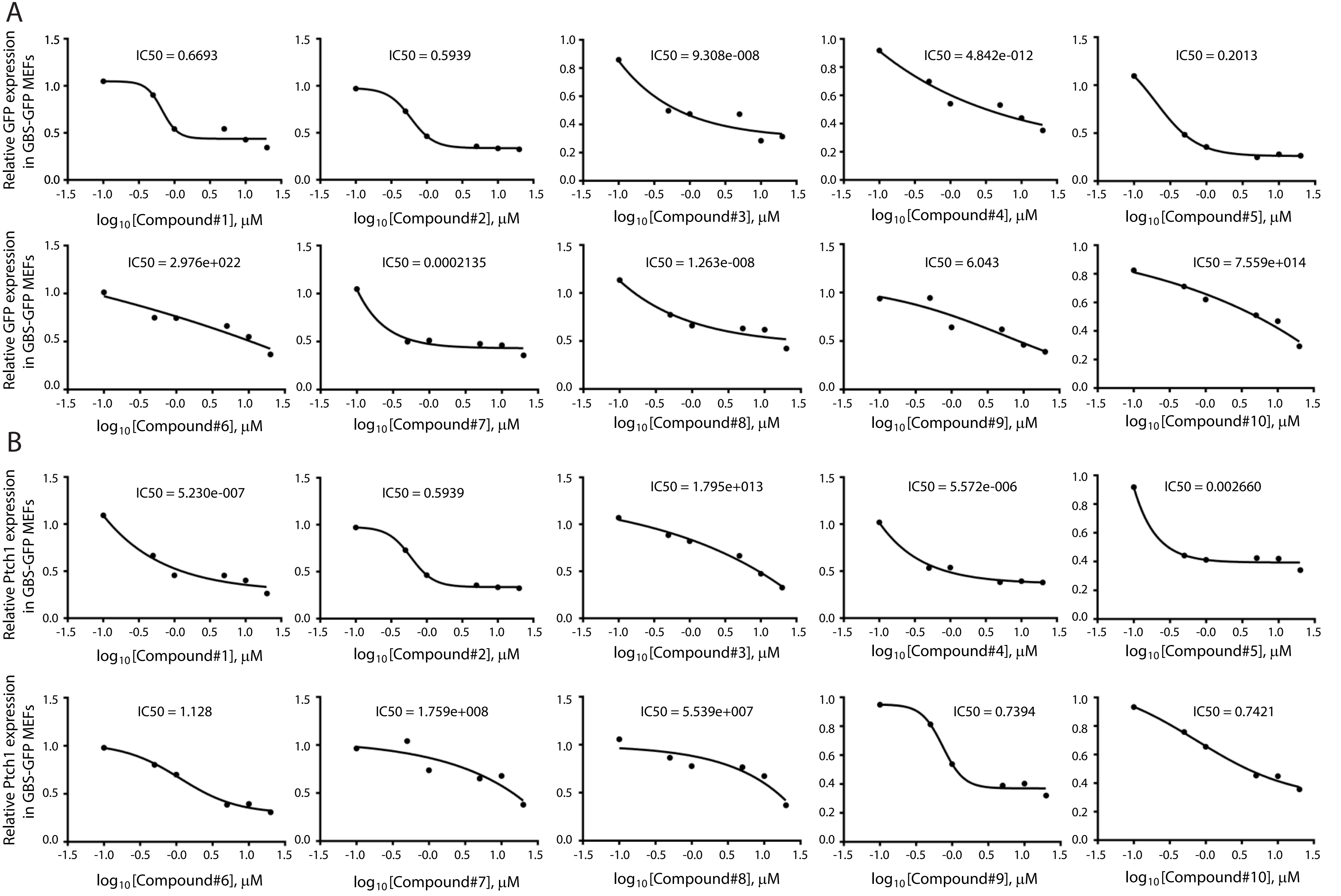
A
Relative GFP expression in GBS-GFP MEFs
IC50 = 0.6693
log10[Compound#1], μM
IC50 = 0.5939
log10[Compound#2], μM
IC50 = 9.308e-008
log10[Compound#3], μM
IC50 = 4.842e-012
log10[Compound#4], μM
IC50 = 0.2013
log10[Compound#5], μM
IC50 = 2.976e+022
log10[Compound#6], μM
IC50 = 0.0002135
log10[Compound#7], μM
IC50 = 1.263e-008
log10[Compound#8], μM
IC50 = 6.043
log10[Compound#9], μM
IC50 = 7.559e+014
log10[Compound#10], μM
B
Relative Ptch1 expression in GBS-GFP MEFs
IC50 = 5.230e-007
log10[Compound#1], μM
IC50 = 0.5939
log10[Compound#2], μM
IC50 = 1.795e+013
log10[Compound#3], μM
IC50 = 5.572e-006
log10[Compound#4], μM
IC50 = 0.002660
log10[Compound#5], μM
IC50 = 1.128
log10[Compound#6], μM
IC50 = 1.759e+008
log10[Compound#7], μM
IC50 = 5.539e+007
log10[Compound#8], μM
IC50 = 0.7394
log10[Compound#9], μM
IC50 = 0.7421
log10[Compound#10], μM

Figure S7.

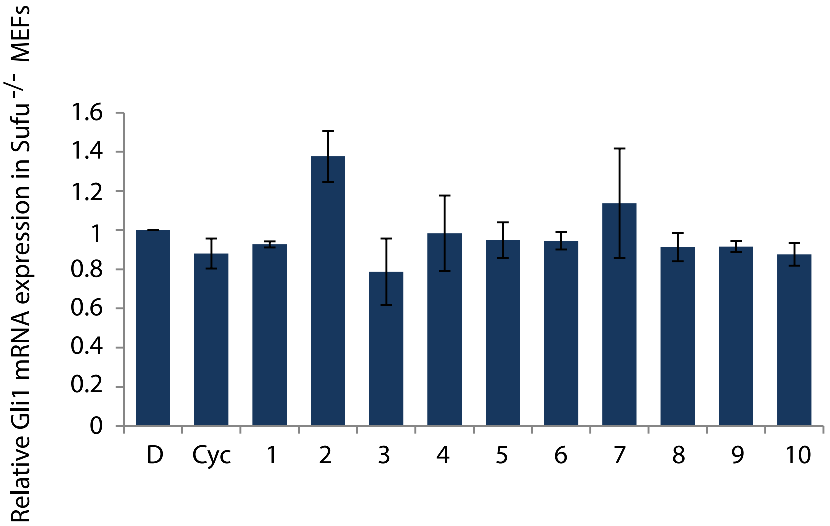
Relative Gli1 mRNA expression in Sufu -/- MEFs
1.6
1.4
1.2
1
0.8
0.6
0.4
0.2
0
D
Cyc
1
2
3
4
5
6
7
8
9
10

Figure S8.

A

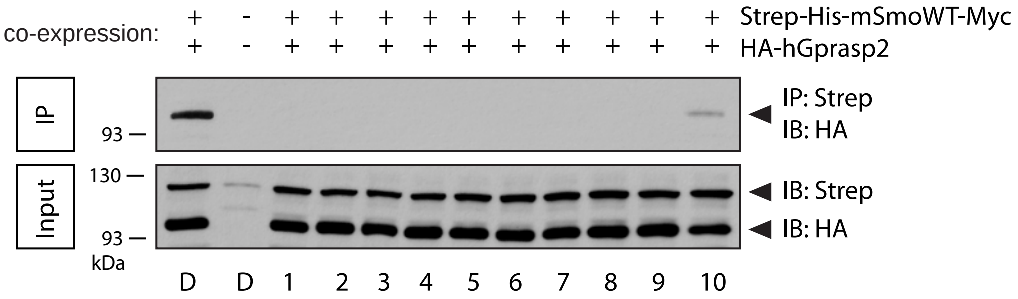
co-expression:
Strep-His-mSmoWT-Myc
HA-hGprasp2
IP
Input
93
130
kDa
IP: Strep
IB: HA
IB: Strep
IB: HA
D D 1 2 3 4 5 6 7 8 9 10

B

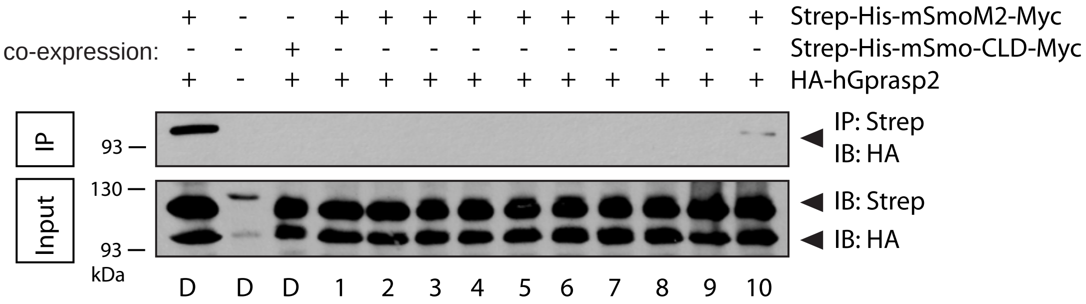
co-expression:
Strep-His-mSmoM2-Myc
Strep-His-mSmo-CLD-Myc
HA-hGprasp2
IP
Input
93
130
kDa
IP: Strep
IB: HA
IB: Strep
IB: HA
D D D 1 2 3 4 5 6 7 8 9 10

Figure S9.

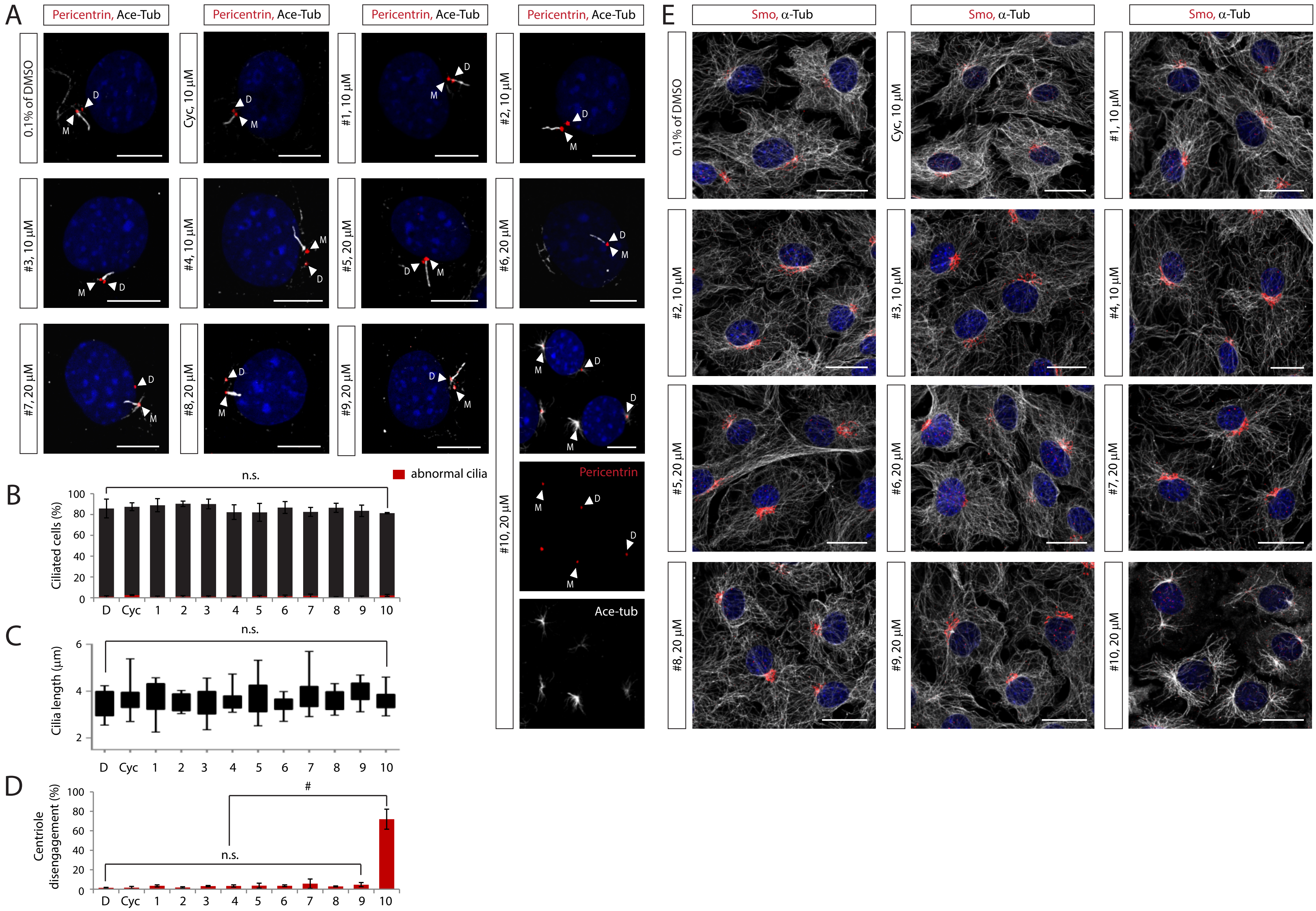

A
Pericentrin, Ace-Tub
0.1% of DMSO
Cyc, 10 μM
#1, 10 μM
#2, 10 μM
#3, 10 μM
#4, 10 μM
#5, 20 μM
#6, 20 μM
#7, 20 μM
#8, 20 μM
#9, 20 μM
#10, 20 μM
D
M
Pericentrin
Ace-tub
B
abnormal cilia
n.s.
Ciliated cells (%)
100
80
60
40
20
0
D
Cyc
1
2
3
4
5
6
7
8
9
10
C
n.s.
Cilia length (μm)
6
4
2
D
#
n.s.
Centriole disengagement (%)
E
Smo, α-Tub

Figure S10.

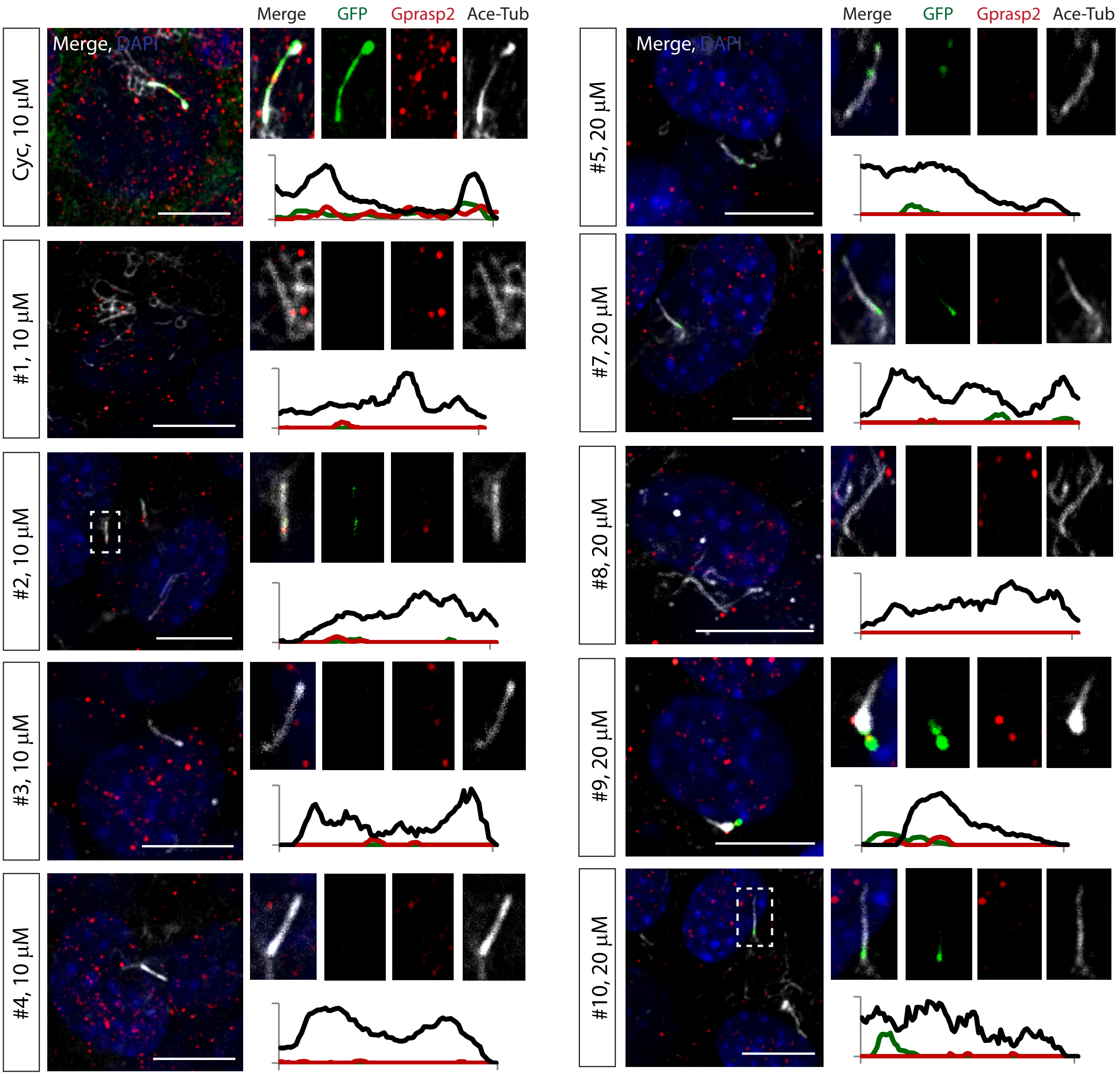

Merge
GFP
Gprasp2
Ace-Tub
Merge, DAPI
Cyc, 10 µM
#1, 10 µM
#2, 10 µM
#3, 10 µM
#4, 10 µM
#5, 20 µM
#7, 20 µM
#8, 20 µM
#9, 20 µM
#10, 20 µM

Figure S11.

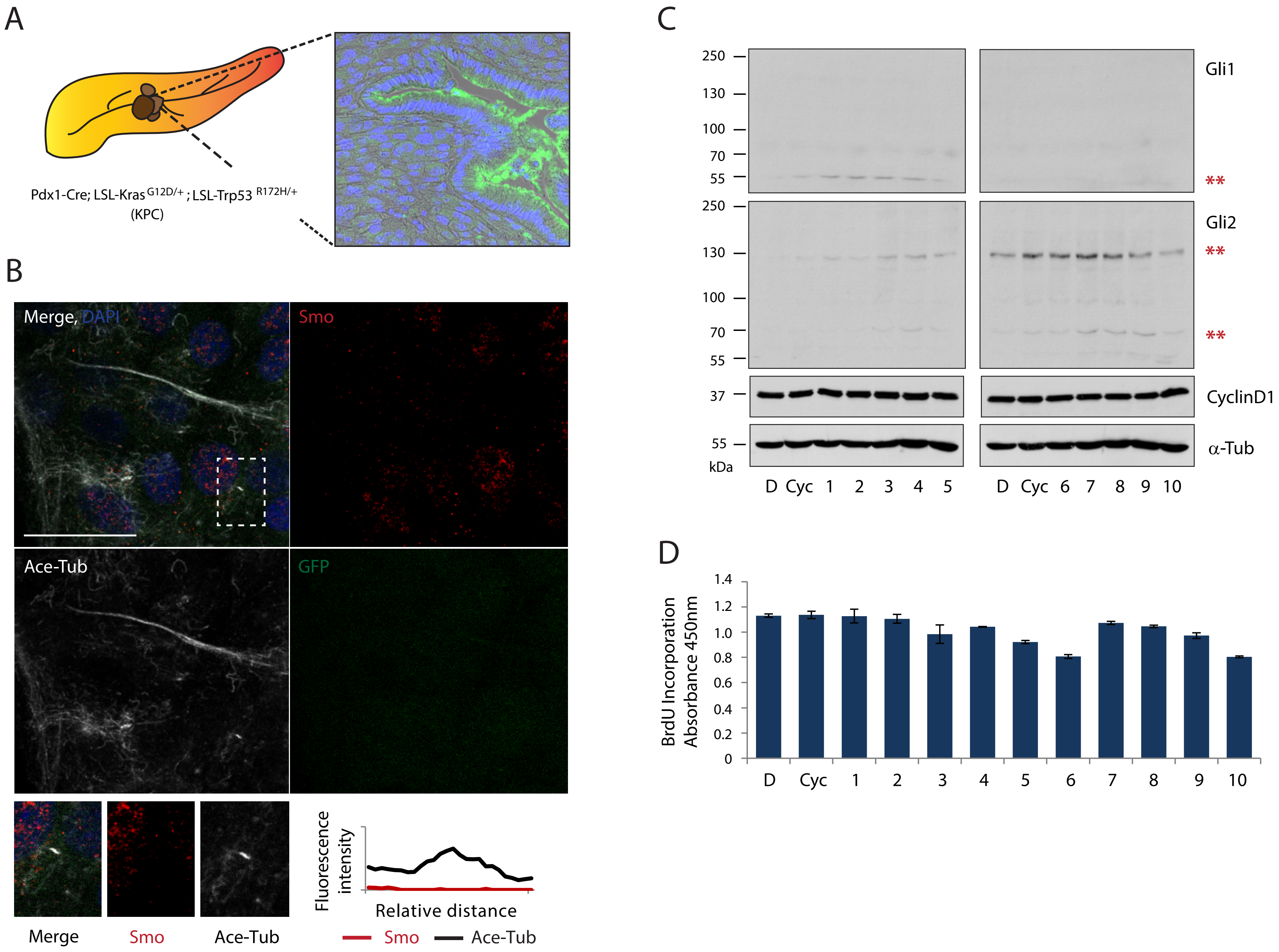
A
Pdx1-Cre; LSL-Kras G12D/+ ; LSL-Trp53 R172H/+
(KPC)
B
Merge, DAPI
Smo
Ace-Tub
GFP
Merge
Smo
Ace-Tub
Fluorescence intensity
Relative distance
Smo
Ace-Tub
C
250
130
100
70
55
250
130
100
70
55
37
55
kDa
Gli1
Gli2
CyclinD1
α-Tub
**
**
**
D Cyc 1 2 3 4 5
D Cyc 6 7 8 9 10
D
BrdU Incorporation
Absorbance 450nm
1.4
1.2
1.0
0.8
0.6
0.4
0.2
0
D Cyc 1 2 3 4 5 6 7 8 9 10

Figure S12.

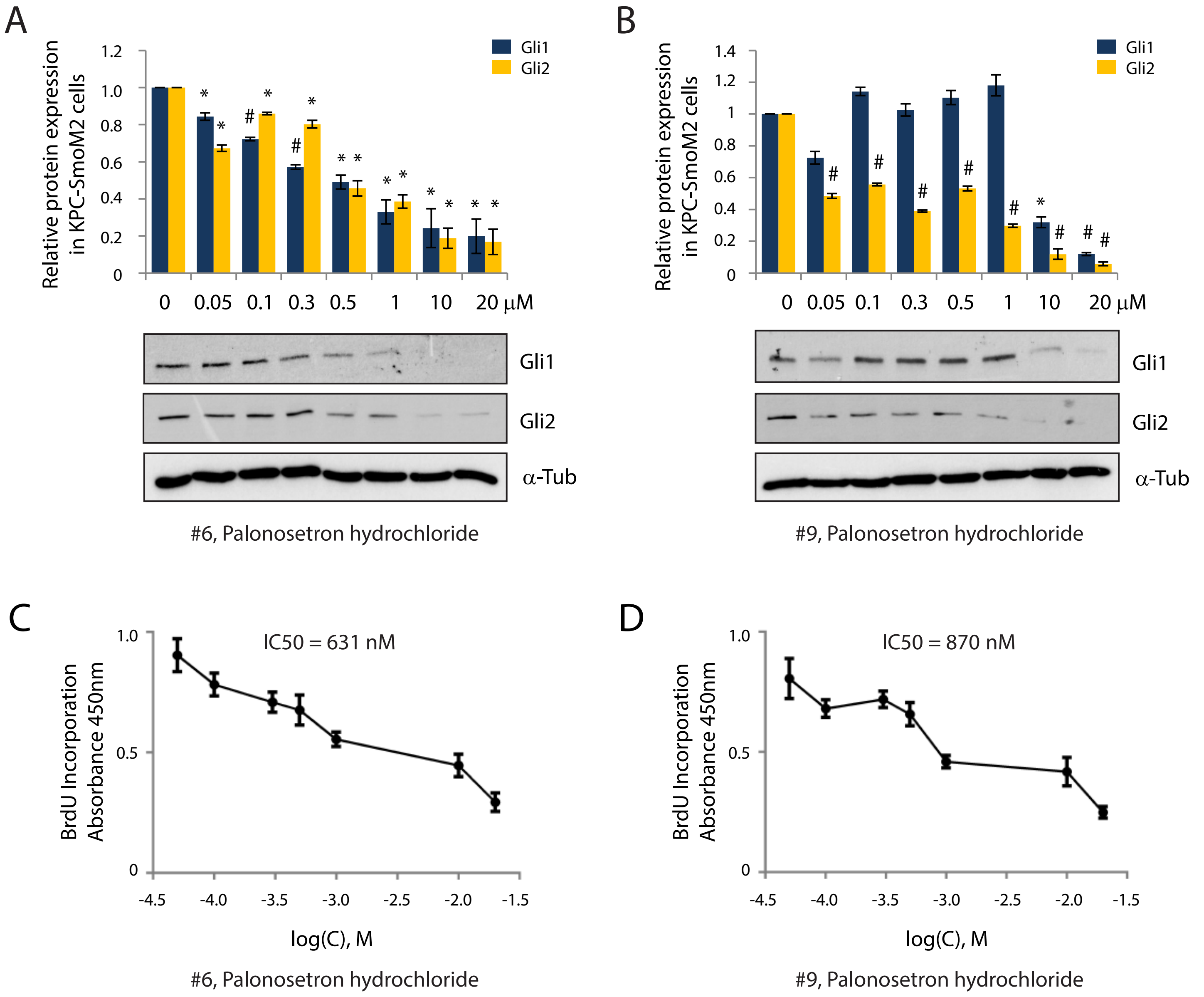
A
Relative protein expression in KPC-SmoM2 cells
Gli1
Gli2
0 0.05 0.1 0.3 0.5 1 10 20 μM
α-Tub
#6, Palonosetron hydrochloride
B
Relative protein expression in KPC-SmoM2 cells
Gli1
Gli2
0 0.05 0.1 0.3 0.5 1 10 20 μM
α-Tub
#9, Palonosetron hydrochloride
C
BrdU Incorporation Absorbance 450nm
IC50 = 631 nM
log(C), M
#6, Palonosetron hydrochloride
D
BrdU Incorporation Absorbance 450nm
IC50 = 870 nM
log(C), M
#9, Palonosetron hydrochloride
